# Image-Based Identification of Ophthalmic Lens Optical Characteristics: A Benchmark of Local Texture Descriptors for Non-Destructive Optical Inspection

**DOI:** 10.3390/s26185747

**Published:** 2026-09-10

**Authors:** Abdelilah Errachidi, Issam El Khadiri, Youssef El Merabet, Mohamed Kas, Yassine Ruichek, Cyril Meurie, Zahid Akhtar

**Affiliations:** 1 SETIME Laboratory, Department of Physics, Ibn Tofail University, Kenitra 14000, Morocco; abdellah.errachidi@uit.ac.ma (A.E.); issam.elkhadirii@gmail.com (I.E.K.); 2 CIAD UMR7533, Université Bourgogne Franche-Comté, UTBM, 90010 Belfort Cedex, France; mohamed.kas@utbm.fr (M.K.); yassine.ruichek@utbm.fr (Y.R.); 3 Univ Gustave Eiffel, COSYS-LEOST, F-59650, Villeneuve d’Ascq, France; cyril.meurie@univ-eiffel.fr; 4 Department of Electrical and Computer Engineering, State University of New York Polytechnic Institute, Utica, NY 13502, USA; akhtarz@sunypoly.edu

**Keywords:** ophthalmic lenses, texture descriptors, LBP-like methods, micro-texture analysis, refractive index, anti-reflective coatings, cross-dataset validation

## Abstract

Despite recent advances in computer vision and optical imaging, the image-based recognition of ophthalmic lens categories characterized by different refractive indices and anti-reflective coating types remains largely unexplored. In this work, we formulate this problem as a non-destructive optical inspection task, where lens-induced visual changes are treated as subtle micro-texture signatures produced by the interaction between the lens and a controlled visual target. Although these signatures are often imperceptible to the human eye, they can be quantified using local texture analysis. To establish the first systematic benchmark for this emerging application, we present a comprehensive evaluation of state-of-the-art local texture descriptors, with particular emphasis on Local Binary Pattern (LBP)-like methods, under unified evaluation protocols. The evaluation is conducted on two dedicated ophthalmic lens image datasets acquired under controlled conditions, using consistent visual targets designed to reveal lens-induced texture and color variations. This setting enables a rigorous assessment of whether handcrafted micro-texture descriptors can capture discriminative image signatures associated with different refractive indices and anti-reflective coating types. The experimental results demonstrate that several modern LBP variants provide excellent recognition performance, confirming the relevance of local micro-texture analysis for image-based ophthalmic lens category recognition. Comparisons with pretrained deep feature extractors further show that although deep representations generally achieve the highest recognition rates, several handcrafted descriptors offer comparable performance while requiring neither network training nor fine-tuning. Beyond recognition accuracy, the benchmark investigates performance stability across datasets, robustness under limited training samples, classifier influence, and the statistical significance of the observed performance differences. Overall, this work establishes the first reproducible image-based benchmark for non-destructive ophthalmic lens inspection and provides practical guidelines for selecting local texture descriptors in ophthalmic lens recognition systems.

## 1. Introduction

Eyeglasses are among the most widely used medical devices worldwide. According to the World Health Organization, more than two billion people rely on corrective glasses on a daily basis [1]. Beyond correcting visual defects such as myopia, hyperopia, astigmatism, or presbyopia, ophthalmic lenses also provide protection against harmful radiation (e.g., ultraviolet rays, blue light) and serve aesthetic as well as ergonomic purposes. Among their intrinsic optical characteristics, the refractive index plays a central role, as it governs the lens material’s ability to bend light and directly impacts thickness, weight, and optical performance. For instance, low-index lenses (e.g., 1.50) are typically thicker and heavier than high-index lenses (e.g., 1.60 or 1.67), which translates into practical benefits for end users in terms of comfort, aesthetics, and reduced chromatic aberrations. Accurate knowledge of a lens’s refractive index is therefore essential for both practitioners and researchers. Yet, determining this parameter in clinical practice remains challenging, as existing techniques (e.g., precision refractometry, ellipsometry, interferometry) require sophisticated and costly instrumentation, often restricted to specialized laboratories. This gap highlights the need for simpler, portable, and automated methods capable of automatically identifying ophthalmic lens categories characterized by different refractive indices and anti-reflective coatings without relying on complex optical setups.

Recent advances in artificial intelligence (AI) and computer vision offer promising alternatives for non-invasive material characterization. In particular, image-based classification methods have demonstrated remarkable success in a wide range of domains, from medical diagnostics [2,3] and autonomous driving [4,5], to biometrics [6,7], remote sensing [8,9], industrial quality control [10,11], and environmental monitoring [12,13]. This shift in focus—inferring material properties rather than recognizing objects—has recently attracted significant interest in computer vision and machine learning [14,15]. Applications span diverse domains: detecting micro-defects on industrial surfaces [16], classifying fabrics through fine-grained textures [17,18], identifying composite phases or treatment effects in material science and microscopy [19,20,21], and authenticating documents by analyzing microstructures on paper or plastic [22,23]. At its core, image classification relies on detecting and analyzing distinctive attributes such as color, shape, texture, or structural details that enable the separation of one class from another. While modern learning paradigms have significantly improved performance in this area, their success still critically hinges on robust feature extraction, accurate pattern interpretation, and the constraints imposed by the target application, particularly in cases where visual variations are subtle or scarcely perceptible.

To the best of our knowledge, no systematic image-based benchmark has yet addressed the recognition of ophthalmic lens categories characterized by different refractive indices and anti-reflective coating types. Although these properties do not visibly alter the structure or semantic content of a scene, they subtly affect how light propagates through the lens, producing nearly imperceptible modifications in the captured image. From a physical perspective, these subtle image variations arise from the combined effects of refractive index, material dispersion, lens geometry, surface reflectance, anti-reflective coating interference, optical aberrations, and the camera acquisition system. Rather than directly measuring these intrinsic optical parameters, local texture descriptors characterize the visual signatures generated by their combined influence on image sharpness, local contrast, spatial-frequency content, and chromatic appearance. Consequently, the proposed framework should be interpreted as an image-based recognition approach that exploits these physically induced visual signatures rather than as a direct measurement of the optical properties of ophthalmic lenses. These visual signatures manifest themselves through local contrast variations, chromatic dispersion, edge definition, image sharpness, color balance, and micro-texture. While the overall appearance of the scene remains unchanged to the human eye, these latent distortions encode valuable information about the lens itself. The analysis of these subtle visual cues opens up a new research direction for the non-invasive identification of optical components, making it possible to distinguish ophthalmic lens categories exhibiting different refractive indices, surface coatings, or glass materials using only visual data. Although often imperceptible to human vision, subtle textural fluctuations can be effectively captured through robust local descriptors. Accordingly, ophthalmic lens recognition can benefit from texture operators that are sensitive to microstructural distortions introduced by the propagation of light through different optical media. Among the most influential approaches, the family of Local Binary Patterns (LBP) [24] has emerged as a fundamental framework for encoding fine-scale textures. The success of LBP stems from its simplicity, efficiency, and independence from dictionary-based learning, which has made it a widely adopted reference in pattern recognition.

The widespread success of LBP across diverse applications has led to the emergence of a large number of variants aimed at enhancing its discriminative capability, robustness, and suitability for non-traditional texture analysis problems. These include medical image analysis [25,26,27], image retrieval [28,29], person identification [30,31], human activity recognition [32], face recognition [33,34], facial expression recognition [35,36], material recognition [37,38], scene interpretation [39], industrial inspection [40], and others. Song et al. [41] proposed the local grouped order and non-local binary pattern (LGONBP) descriptor for texture classification. LGONBP combines two complementary operators: the LGOP component groups neighboring pixels with respect to a dominant direction and encodes their order relationships, while the NLBP component captures long-range intensity differences using anchors derived from global image statistics. Their integration, reinforced by central pixel encoding, yields discriminative histogram features for robust texture classification. Naser et al. [26] developed a distinct family of LBP descriptors based on local cumulative pixel disparities for X-ray pneumonia detection. The descriptors were designed using a circular neighborhood adapted from the original square filter, thereby accounting for both angular and radial variations present in the micro- and macro-textures of the images. Finally, Garg et al. [25] formulated the wavelet-based shifted elliptical local binary pattern (WSELBP) for early Alzheimer’s disease detection. Their approach applies the double-density dual tree complex wavelet transform (DD-DTCWT) to MRI scans to generate sixteen directional sub-bands, over which shifted elliptical local binary patterns (ELBP) are computed. This combination enables the extraction of multi-scale directional micro- and macro-patterns, effectively capturing grey matter and structural changes at different disease stages.

## 2. Motivation

For more than a decade, LBP-like methods have dominated research on local feature extraction, and the study of local descriptors in pattern recognition remains highly active, as evidenced by the large number of recent works in this area. The need for robust local descriptors with strong discriminative power is well recognized, and numerous powerful LBP variants continue to be developed in the literature. Recent contributions include PGMO-MSTP (petersen graph multi-orientation based multi-scale ternary pattern) [42], MDGMM-LTP (multi-directional guided mixed mask local ternary pattern) [43], CS-CoLBP (cross-scale co-occurrence local binary pattern) [44], LDDRD (local directional difference and relational descriptor) [45], SARLBP (scale-adaptive robust LBP) [46], FDOG-LBP-M (fractional derivative-based LBP method) [47], RGP (regional gradient pattern) [48], HLBP (hexagonal LBP) [29], wavelet-based multiscale captioning descriptors [49], QS-LBP (quad-star LBP) [27], and LRGIOP (local radial grouped invariant order pattern) [50], among others.

The vast majority of existing texture descriptors, and LBP variants in particular, are generally conceived—or at least tailored—for specific applications. As a result, they may not always be well-suited to the challenges posed by other tasks or capable of handling the full range of irregularities that can occur in images. Furthermore, the growing demand for discriminative and robust descriptors across a wide range of applications naturally raises the question of suitability: how well can a given LBP-like descriptor address the requirements of a particular application? In light of these considerations, and following previous works, application-oriented comparative studies on the effectiveness of LBP variants have gained increasing relevance and attention.

Several studies in the literature have focused on reviewing the applicability of LBP-like methods across different application domains:

- To establish a general-purpose framework for texture analysis, Fernandez et al. [51] implemented 38 LBP variants and conducted experiments on eleven texture datasets. The study reveals consistent performance trends, indicating the superiority of multi-level discretization over simple binarization, the advantage of parametric over non-parametric methods, the positive impact of higher dimensionality, and the greater effectiveness of point-to-average thresholding compared to point-to-point thresholding.
- Liu et al. [52] reviewed and compared 40 approaches, including both LBP-based and non-LBP methods, categorizing and evaluating them across thirteen texture datasets. The study highlights that the effectiveness of LBP variants is highly dataset-dependent. These large-scale comparative evaluations not only expose their respective strengths and weaknesses but also serve as a valuable guideline for researchers and practitioners in selecting the most appropriate descriptors according to specific application requirements. In this work, MRELBP (Median Robust Extended Local Binary Pattern) is shown to achieve stronger discriminative power by spanning a larger spatial domain through its sampling scheme and by applying thresholding on local medians rather than raw pixel values, which significantly enhances robustness.

More recently:

- Slimani et al. [53] conducted a review of 22 state-of-the-art LBP-like descriptors along with some of their recent variants, and presented a comparative analysis for facial expression recognition using two benchmark databases. In this work, it is highlighted that descriptors based on dominating set and graph theory exhibit significant performance stability, as they consistently rank among the best-performing LBP variants across the evaluated databases.
- El Idrissi et al. [54] compared and evaluated 50 LBP variants and non-LBP texture methods on the palmprint recognition problem using five datasets. The study reveals that filtering-based approaches, together with methods relying on neighborhood topology and sampling, as well as descriptors founded on dominating set and graph theory, exhibit remarkable performance stability across a wide range of palmprint datasets.
- The work presented in [55] represents the first attempt to evaluate the potential of texture descriptors in classifying benthic species and habitats using challenging underwater imagery from the SE Baltic Sea reefs. The results reveal that the MRELBP algorithm achieved the highest overall performance. The authors noted that, although the MRELBP descriptor outperformed the other algorithms in most classes, it still encountered challenges in distinguishing morphologically and visually similar species, such as the filamentous algae *V. fucoides* and *Cladophora* sp.

As evidenced by prior works, notable limitations persist in existing application-oriented comparisons of LBP-like descriptors. Their results consistently show strong dataset- and task-dependence, indicating that no single descriptor is universally superior across all applications and benchmark datasets. Consequently, selecting an appropriate descriptor for a specific real-world setting remains challenging, especially when the target visual signatures are subtle and application-dependent. Moreover, many existing surveys and comparative studies are limited by restricted descriptor coverage, heterogeneous evaluation protocols, or datasets that do not reflect the specific characteristics of emerging inspection tasks. To the best of our knowledge, the image-based recognition of ophthalmic lens characteristics associated with different refractive indices and anti-reflective coating types has not yet been systematically investigated through a dedicated benchmark of local texture descriptors. This paper addresses this gap by providing a systematic and up-to-date comparative evaluation of traditional and recent LBP-like texture descriptors for non-destructive ophthalmic lens inspection. The study focuses on plano lenses with three anti-reflective coating categories, namely green, blue, and white organic coatings, and four commonly used refractive indices, namely 1.50, 1.56, 1.60, and 1.67. No geometric or photometric corrections are applied, in order to evaluate the descriptors under a direct image-based acquisition setting. The main contributions of this study are summarized as follows:

- Two original ophthalmic lens image datasets, namely TexLensDB and ColLensDB, are introduced to study subtle lens-induced visual variations associated with refractive index and anti-reflective coating type. The datasets are acquired under controlled imaging conditions using fixed visual targets and constitute the first benchmark for image-based ophthalmic lens recognition.
- A comprehensive and fair benchmark of a large collection of traditional and recent local texture descriptors is conducted under unified experimental protocols, including both grayscale and color-based methods.
- Representative pretrained CNN- and Transformer-based feature extractors are evaluated under exactly the same experimental protocol, enabling a direct comparison between handcrafted and deep visual representations.
- The proposed benchmark investigates descriptor performance under standard and low-shot training regimes, analyzes the influence of different distance metrics and classifier settings, and provides a statistical analysis of the observed performance differences.
- The resulting benchmark provides practical guidelines for selecting suitable handcrafted and deep feature representations for non-destructive ophthalmic lens recognition.

The rest of this paper is organized as follows. Section 3 presents a literature review of local texture descriptors, with emphasis on LBP-like methods and their representative extensions. Section 4 describes the construction of TexLensDB and ColLensDB datasets. Section 5 reports the experimental results under standard and low-shot regimes and discusses the main performance trends observed across the evaluated descriptors. Finally, Section 6 summarizes the main findings and outlines future research directions.

## 3. Literature Review

Texture feature extraction is a fundamental step in image recognition systems, as the discriminative power of the extracted features directly influences the relevance and reliability of the recognition results. Even when an efficient classifier is used, the overall classification performance may remain limited if the extracted features do not capture sufficiently meaningful visual information. Feature extraction can therefore be viewed as a compact representation process, in which raw image data are transformed into a set of informative descriptors. An effective descriptor should preserve the relevant texture signatures of the images while reducing redundant or irrelevant information, thereby providing a suitable compromise between accuracy, robustness, and computational cost.

In this section, we first introduce the formal notation used to compute the conventional Local Binary Pattern (LBP) operator, describe its encoding mechanism, and summarize its main characteristics. We then review several representative state-of-the-art local texture descriptors that are relevant to the application addressed in this paper. The objective is to assess their ability to capture subtle lens-induced visual distortions for the image-based identification of ophthalmic lens characteristics.

### 3.1. Local Binary Pattern (LBP)

The Local Binary Pattern (LBP), introduced by Ojala et al. [24], is a cornerstone descriptor for modeling local micro-structures in intensity images. It compares a central pixel $I_{c}$ with its *P* neighboring pixels ${\left\{I_{p}\right\}}_{p=0}^{P-1}$, sampled on a circle of radius *R*. By applying a binary threshold to the differences $I_{p}-I_{c}$, a *P*-bit code is generated for each central pixel. The resulting codes are then accumulated into a histogram over the image to form a compact texture descriptor. Formally, for a given central pixel $I_{c}$, the LBP code is computed as follows:

(1) $${\mathrm{LBP}}_{P,R}\left(I_{c}\right)=\sum_{p=0}^{P-1}b\left(I_{p}-I_{c}\right)\;2^{p},$$

where b(·) is the binary thresholding function defined by

(2) $$b\left(x\right)=\left\{\begin{array}{cc} 1, & x\geq 0,\\ 0, & x<0, \end{array}\right.$$

As a result, the LBP operator produces a local code that is relatively stable under monotonic intensity changes and is able to characterize basic micro-structures such as edges, corners, line endings, and flat areas. However, despite its simplicity and effectiveness, conventional LBP has several drawbacks. In particular, it may lead to high-dimensional histograms, remains sensitive to image rotation, and can be affected by noise or small local intensity fluctuations.

**Figure 1:**
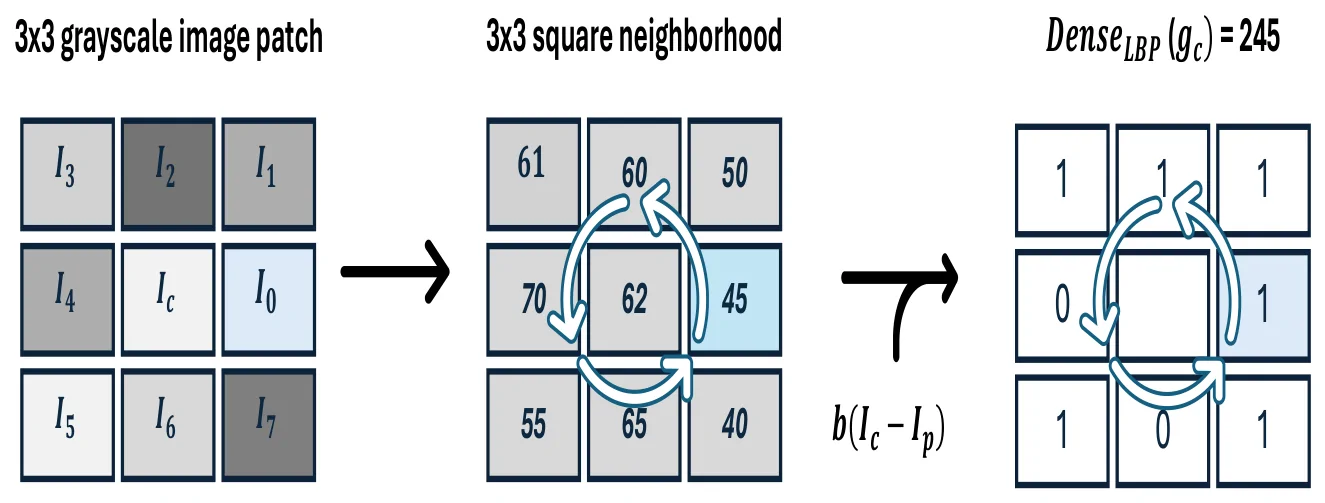
Dense LBP on a 3×3 neighborhood (P=8,R=1). Neighbors $I_{0},\ldots ,I_{7}$ (traversed clockwise from $I_{0}$) are thresholded against $I_{c}$; the resulting 8-bit string is converted to an integer code. Dense evaluation plus histogramming yields a compact global texture descriptor.

Figure 1 shows the dense LBP encoding mechanism on a 3×3 neighborhood. In the context of ophthalmic lens characteristic identification, such local encoding is useful because it summarizes fine micro-structural variations that may be induced by subtle optical distortions.

### 3.2. Quad Star Local Binary Pattern (QS-LBP)

**Figure 2:**
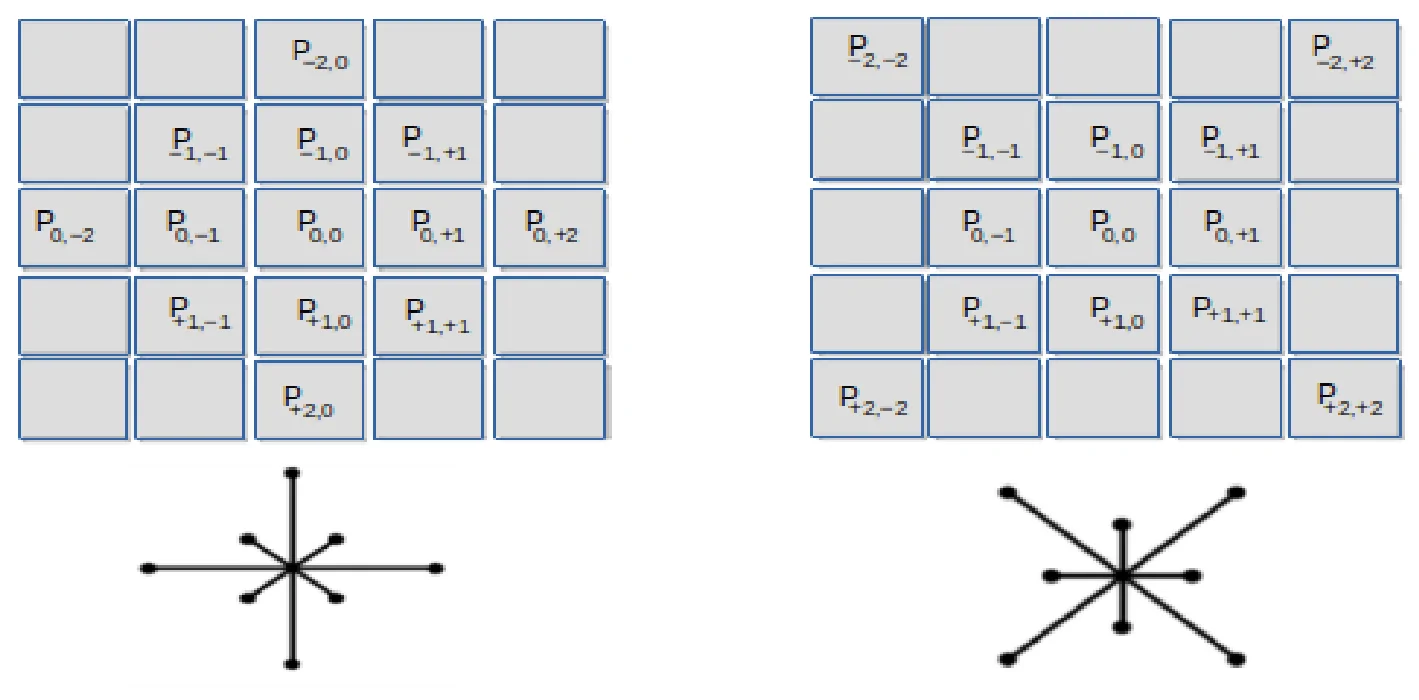
Architecture of the two models adopted in the QS-LBP method.

Quad Star Local Binary Pattern (QS-LBP) was proposed by Gül [27] as an LBP-based feature extraction method for breast cancer histopathology image analysis. The method extends the conventional LBP principle by replacing the standard 3×3 neighborhood with a 5×5 local patch. Instead of comparing each neighbor directly with the central pixel, QS-LBP compares selected pairs of pixels arranged according to star-like configurations around the center. This structure gives the descriptor its name, Quad Star LBP. Let $P_{i,j}$ denote the gray-level value of the pixel located at relative position (i,j) with respect to the central pixel of a 5×5 patch. QS-LBP defines two local encoding models. Figure 2 illustrates the architectural structure of the QS-LBP method. In the first model, the selected pixels follow a star-like path around the central position. The binary pattern is computed as

(3) $$\begin{array}{cc} P_{c}^{\left(1\right)}= & \;(b\left(P_{-1,-1}-P_{-2,0}\right),\;b\left(P_{-2,0}-P_{-1,+1}\right),\\ \; & b\left(P_{-1,+1}-P_{0,+2}\right),\;b\left(P_{0,+2}-P_{+1,+1}\right),\\ \; & b\left(P_{+1,+1}-P_{+2,0}\right),\;b\left(P_{+2,0}-P_{+1,-1}\right),\\ \; & b\left(P_{+1,-1}-P_{0,-2}\right),\;b\left(P_{0,-2}-P_{-1,-1}\right)). \end{array}$$

The resulting binary sequence is then converted into a decimal code as follows:

(4) $$\mathrm{QS}\text{-}{\mathrm{LBP}}^{\left(1\right)}=\sum_{k=0}^{7}P_{c}^{\left(1\right)}\left(k\right)\;2^{k},$$

where $P_{c}^{\left(1\right)}\left(k\right)$ denotes the *k*-th binary value generated by the first model.

The second model uses another star-like sampling configuration within the same 5×5 patch. Its binary pattern is defined by

(5) $$\begin{array}{cc} P_{c}^{\left(2\right)}= & \;(b\left(P_{-2,-2}-P_{-1,0}\right),\;b\left(P_{-1,0}-P_{-2,+2}\right),\\ \; & b\left(P_{-2,+2}-P_{0,+1}\right),\;b\left(P_{0,+1}-P_{+2,+2}\right),\\ \; & b\left(P_{+2,+2}-P_{+1,0}\right),\;b\left(P_{+1,0}-P_{+2,-2}\right),\\ \; & b\left(P_{+2,-2}-P_{0,-1}\right),\;b\left(P_{0,-1}-P_{-2,-2}\right)). \end{array}$$

Similarly, the corresponding decimal code is obtained by

(6) $$\mathrm{QS}\text{-}{\mathrm{LBP}}^{\left(2\right)}=\sum_{k=0}^{7}P_{c}^{\left(2\right)}\left(k\right)\;2^{k}.$$

Thus, QS-LBP generates two local binary patterns from two different star-shaped configurations. By considering pixel relationships over a 5×5 neighborhood, QS-LBP captures longer-range local spatial transitions than conventional LBP, which is generally restricted to immediate neighbors. This makes it suitable for representing local micro-structural variations.

### 3.3. Radial Cross Symmetric Pattern (RCSP)

**Figure 3:**
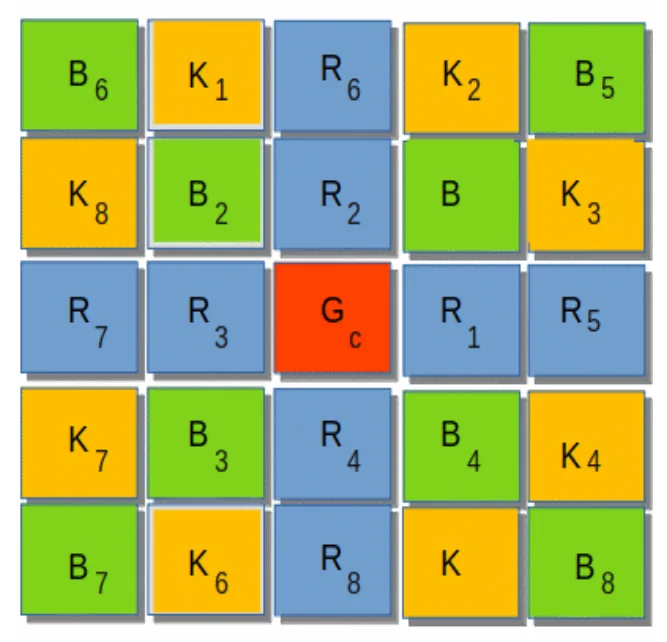
Numbering scheme of chessmen followed for feature extraction through Modified Chess Patterns.

Radial Cross Symmetric Pattern (RCSP) was proposed by Kartheek et al. [56] as a handcrafted local texture descriptor for facial expression recognition. The method was introduced together with two intermediate descriptors, namely Radial Cross Pattern (RCP) and Chess Symmetric Pattern (CSP), and is computed on a 5×5 local neighborhood. The main idea is to exploit both neighboring-pixel relationships and adjacent-pixel relationships in order to capture fine local texture variations. In a 5×5 patch, the 24 pixels surrounding the central pixel are divided into two groups: RCP uses 16 pixels and generates two feature values, whereas CSP uses the remaining 8 pixels and generates one feature value. The fusion of RCP and CSP forms the final RCSP descriptor. Let $G_{c}$ denote the gray-level value of the central pixel in a 5×5 neighborhood. To enhance the representation of texture details, the numbering scheme is defined in two directions: clockwise for CSP and anti-clockwise for RCP. The numbering assigned to the Rook, Bishop, and Knight positions is shown in Figure 3. The RCP component is divided into two sub-patterns, denoted by ${\mathrm{RCP}}_{1}$ and ${\mathrm{RCP}}_{2}$. The first sub-pattern, ${\mathrm{RCP}}_{1}$, compares four Rook-position pixels from the 3×3 neighborhood and four Bishop-position pixels from the 5×5 neighborhood with the central pixel $G_{c}$. It is defined by

(7) $$\begin{array}{cc} {\mathrm{RCP}}_{1}=\{ & \;b\left(R_{1}-G_{c}\right),b\left(R_{2}-G_{c}\right),b\left(R_{3}-G_{c}\right),b\left(R_{4}-G_{c}\right),\\ \; & \;b\left(B_{5}-G_{c}\right),b\left(B_{6}-G_{c}\right),b\left(B_{7}-G_{c}\right),b\left(B_{8}-G_{c}\right)\}. \end{array}$$

The second sub-pattern, ${\mathrm{RCP}}_{2}$, compares four Bishop-position pixels from the 3×3 neighborhood and four Rook-position pixels from the 5×5 neighborhood with $G_{c}$

(8) $$\begin{array}{cc} {\mathrm{RCP}}_{2}=\{ & \;b\left(B_{1}-G_{c}\right),b\left(B_{2}-G_{c}\right),b\left(B_{3}-G_{c}\right),b\left(B_{4}-G_{c}\right),\\ \; & \;b\left(R_{5}-G_{c}\right),b\left(R_{6}-G_{c}\right),b\left(R_{7}-G_{c}\right),b\left(R_{8}-G_{c}\right)\}. \end{array}$$

Each binary pattern can be converted into a numerical code by multiplying it with a weight vector $W_{z}$, where different weighting schemes can be used, such as binary, Fibonacci, prime, natural, square, odd, or even weights

(9) $${\mathrm{RCP}}_{1}=\sum \left({\mathrm{RCP}}_{1}\times W_{z}\right),\;{\mathrm{RCP}}_{2}=\sum \left({\mathrm{RCP}}_{2}\times W_{z}\right).$$

The complete Radial Cross Pattern is then obtained by concatenating the two sub-patterns

(10) $$\mathrm{RCP}={\mathrm{RCP}}_{1}\cup {\mathrm{RCP}}_{2}.$$

The CSP component is inspired by center-symmetric comparisons and uses the Knight-position pixels in the 5×5 neighborhood. It compares adjacent Knight-position pairs in horizontal, vertical, and diagonal directions

(11) $$\begin{array}{cc} \mathrm{CSP}=\{ & \;b\left(K_{1}-K_{5}\right),b\left(K_{2}-K_{6}\right),b\left(K_{3}-K_{7}\right),b\left(K_{4}-K_{8}\right),\\ \; & \;b\left(K_{1}-K_{6}\right),b\left(K_{2}-K_{5}\right),b\left(K_{3}-K_{8}\right),b\left(K_{4}-K_{7}\right)\}. \end{array}$$

Similarly, the CSP code is obtained using the selected weight vector

(12) $$\mathrm{CSP}=\sum \left(\mathrm{CSP}\times W_{z}\right).$$

Finally, the RCSP descriptor is obtained by concatenating the RCP and CSP components

(13) RCSP=RCP∪CSP.

Thus, RCSP combines radial cross information and center-symmetric Knight-position relationships in a 5×5 neighborhood. By integrating RCP and CSP, the descriptor captures both multi-radial and multi-orientation local structures. In the original study, RCSP was applied to facial expression recognition and evaluated on several facial expression datasets. Its design is particularly useful for capturing fine local appearance changes, since it analyzes a wider neighborhood than conventional 3×3 LBP while preserving a handcrafted and non-parametric feature extraction framework.

### 3.4. Angular and Radial Local Binary Patterns (ARLBP)

**Figure 4:**
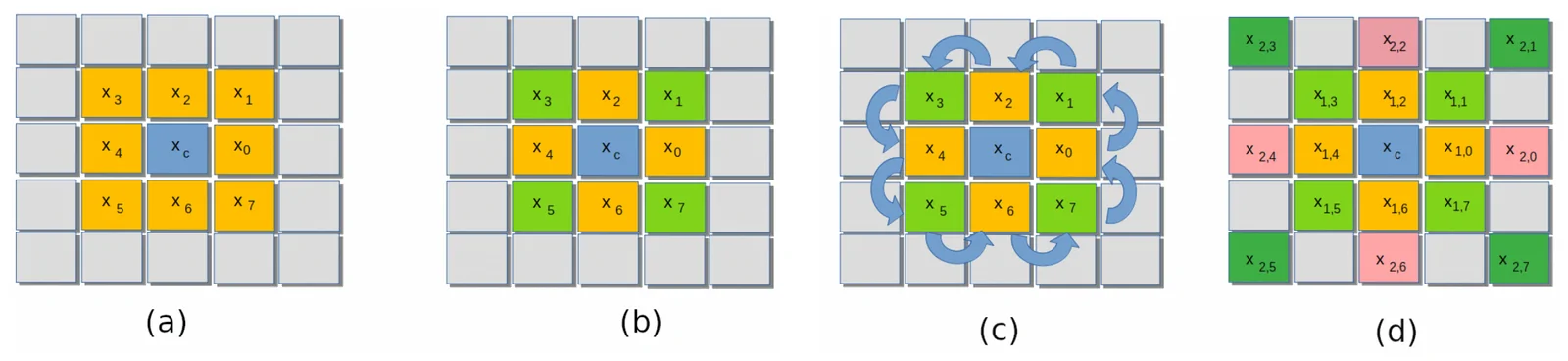
Illustration of (**a**) LBP_SQR calculation, (**b**) LBP_CIR calculation, (**c**) LBP_AD calculation and (**d**) LBP_RD calculation.

Angular and Radial Local Binary Patterns (ARLBP) were proposed by Zaeri and Dib [26] for pneumonia detection from chest X-ray images. The method extends the conventional LBP operator by introducing a family of LBP-like descriptors based on circular neighborhoods, angular differences, and radial differences. The main motivation is that standard LBP captures only very local intensity variations and may fail to represent larger spatial transitions or more complex texture structures. ARLBP therefore aims to encode both micro-structural and macro-structural texture changes by analyzing local pixel relationships along angular and radial directions. Let $x_{c}$ denote the central pixel and let ${\left\{x_{n}\right\}}_{n=0}^{p-1}$ be the set of p=8 neighboring pixels. The classical square LBP is first defined as (cf. Figure 4a)

(14) $${\mathrm{LBP}}_{\mathrm{SQR}}=\sum_{n=0}^{p-1}b\left(x_{n}^{\mathrm{SQR}}-x_{c}\right)\;2^{n},$$

where $x_{n}^{\mathrm{SQR}}$ denotes the neighboring pixels in the conventional square neighborhood.

The first extension introduced in ARLBP is the circular LBP descriptor. It is computed by replacing the original square neighborhood with a circular neighborhood (cf. Figure 4b)

(15) $${\mathrm{LBP}}_{\mathrm{CIR}}=\sum_{n=0}^{p-1}b\left(x_{n}^{\mathrm{CIR}}-x_{c}\right)\;2^{n}.$$

Here, $x_{n}^{\mathrm{CIR}}$ represents the pixel values on the circular neighborhood. For horizontal and vertical positions, the corresponding pixels are directly selected, whereas diagonal positions are approximated by averaging the closest horizontal and vertical neighboring pixels. To further capture the relative importance of local intensity deviations with respect to the neighborhood average, an average-based circular LBP is defined as

(16) $${\mathrm{LBP}}_{\mathrm{CIR}\_\mathrm{AVG}}=\sum_{n=0}^{p-1}b\left(\left|x_{n}^{\mathrm{CIR}}-x_{c}\right|-\mu_{\mathrm{CIR}}\right)2^{n},$$

where

(17) $$\mu_{\mathrm{CIR}}=\frac{1}{p}\sum_{n=0}^{p-1}\left|x_{n}^{\mathrm{CIR}}-x_{c}\right|.$$

The angular component of ARLBP measures transitions between consecutive neighbors located on the same circular ring. The angular difference vector is defined as

(18) $$\Delta {\mathrm{Ang}}_{p}={\left[\Delta {\mathrm{Ang}}_{0},\ldots ,\Delta {\mathrm{Ang}}_{p-1}\right]}^{T},$$

with

(19) $$\Delta {\mathrm{Ang}}_{n}=x_{n}-x_{n+1},\;n=0,1,\ldots ,7.$$

The corresponding angular LBP descriptor is then computed as (cf. Figure 4c)

(20) $${\mathrm{LBP}}_{\mathrm{AD}}=\sum_{n=0}^{p-1}b\left(\Delta {\mathrm{Ang}}_{n}\right)\;2^{n}.$$

An average-based angular version is also defined by

(21) $${\mathrm{LBP}}_{\mathrm{AD}\_\mathrm{AVG}}=\sum_{n=0}^{p-1}b\left(\left|\Delta {\mathrm{Ang}}_{n}\right|-\mu_{\mathrm{AD}}\right)2^{n},$$

where

(22) $$\mu_{\mathrm{AD}}=\frac{1}{p}\sum_{n=0}^{p-1}\left|\Delta {\mathrm{Ang}}_{n}\right|.$$

The radial component measures transitions between pixels located in the same direction but on two concentric circular rings. The radial difference vector is given by

(23) $$\Delta {\mathrm{Rad}}_{p}={\left[\Delta {\mathrm{Rad}}_{0},\ldots ,\Delta {\mathrm{Rad}}_{p-1}\right]}^{T},$$

where

(24) $$\Delta {\mathrm{Rad}}_{n}=x_{r,n}-x_{r+1,n},\;n=0,1,\ldots ,7.$$

Thus, the radial LBP descriptor is defined as (cf. Figure 4d)

(25) $${\mathrm{LBP}}_{\mathrm{RD}}=\sum_{n=0}^{p-1}b\left(\Delta {\mathrm{Rad}}_{n}\right)\;2^{n}.$$

Similarly, its average-based version is computed by

(26) $${\mathrm{LBP}}_{\mathrm{RD}\_\mathrm{AVG}}=\sum_{n=0}^{p-1}S\left(\left|\Delta {\mathrm{Rad}}_{n}\right|-\mu_{\mathrm{RD}}\right)2^{n},$$

where

(27) $$\mu_{\mathrm{RD}}=\frac{1}{p}\sum_{n=0}^{p-1}\left|\Delta {\mathrm{Rad}}_{n}\right|.$$

In total, the ARLBP framework combines seven descriptors: ${\mathrm{LBP}}_{\mathrm{SQR}}$, ${\mathrm{LBP}}_{\mathrm{CIR}}$, ${\mathrm{LBP}}_{\mathrm{CIR}\_\mathrm{AVG}}$, ${\mathrm{LBP}}_{\mathrm{AD}}$, ${\mathrm{LBP}}_{\mathrm{AD}\_\mathrm{AVG}}$, ${\mathrm{LBP}}_{\mathrm{RD}}$, and ${\mathrm{LBP}}_{\mathrm{RD}\_\mathrm{AVG}}$. The original method integrates these representations through a voting-based fusion strategy, where the final class decision is obtained by combining the decisions of the individual descriptors. The strength of ARLBP lies in its ability to capture complementary texture information. The circular LBP component describes local circular micro-patterns, the angular component models transitions along the same neighborhood ring, and the radial component captures relationships between concentric neighborhoods. This makes ARLBP suitable for applications where texture changes appear at different local directions and spatial scales.

### 3.5. Dual Neighborhood Thresholding Patterns Based on Directional Sampling (DNTPDS)

**Figure 5:**
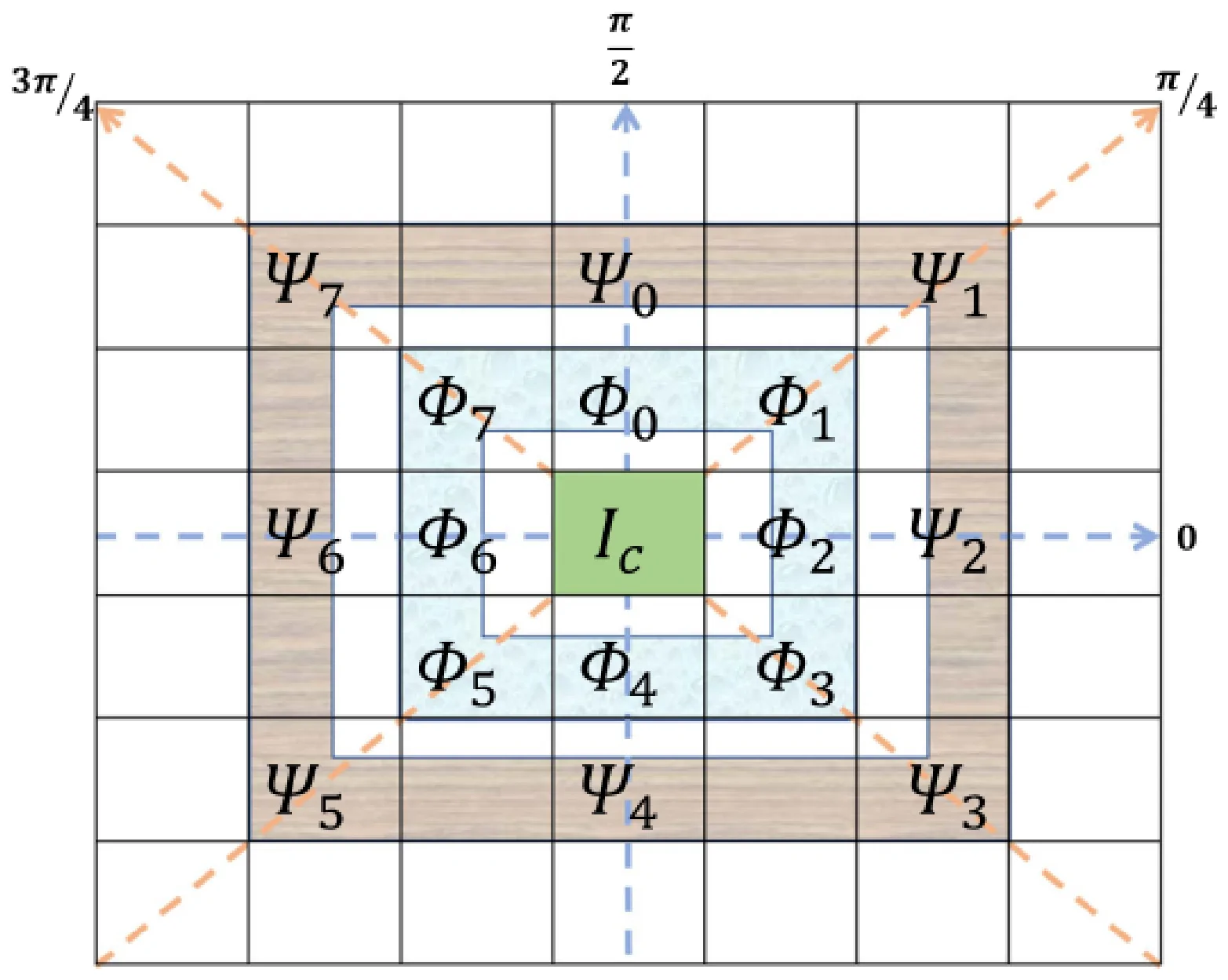
Neighborhood topology of DNTPDS.

Dual Neighborhood Thresholding Patterns based on Directional Sampling (DNTPDS) was proposed by Kas et al. [57] as a handcrafted LBP-like descriptor for face recognition. The descriptor is designed to improve the discriminative power of conventional LBP while keeping a relatively low computational cost. DNTPDS operates on a 5×5 local patch and selects only 16 prominent pixels out of the 25 available pixels (cf. Figure 5). These selected pixels are distributed over two radii, R=1 and R=2, and along four main directions, namely $0^{\circ }$, $45^{\circ }$, $90^{\circ }$, and $135^{\circ }$. Let $I_{c}$ denote the central pixel of a 5×5 grayscale patch. The selected neighboring pixels are divided into two circular groups: $\Phi_{i}$, corresponding to the inner circle of radius R=1, and $\Psi_{i}$, corresponding to the outer circle of radius R=2, with i=0,…,7. The DNTPDS topology therefore combines directional sampling and dual-radius information in order to capture local variations at different spatial distances from the central pixel. The two directional sampling sets used in DNTPDS are defined as

(28) $$\Omega_{1}=\left\{\Phi_{2i}\cup \Psi_{2i+1},\;i=0,\ldots ,3\right\},$$

and

(29) $$\Omega_{2}=\left\{\Phi_{2i+1}\cup \Psi_{2i},\;i=0,\ldots ,3\right\}.$$

The first set combines inner pixels located along the horizontal and vertical directions with outer pixels located along the diagonal directions, whereas the second set uses the opposite configuration. This design allows DNTPDS to encode complementary directional information over two radii. Unlike conventional LBP, which compares neighboring pixels with the central pixel, DNTPDS performs pairwise comparisons between selected pixels from the two directional sampling sets. The DNTPDS code associated with a local patch χ is computed as

(30) $$\Gamma_{\mathrm{DNTPDS}}\left(\chi \right)=\sum_{i=0}^{3}b\left(\Psi_{2i}-\Psi_{2i+1}\right)\;2^{i}+\sum_{i=4}^{7}b\left(\Phi_{2i}-\Phi_{2i+1}\right)\;2^{i}.$$

This formulation produces an 8-bit code, as in conventional LBP, but it is computed from a richer 5×5 neighborhood. The lower binary weights are assigned to comparisons on the outer circle, whereas the higher binary weights are assigned to comparisons on the inner circle. After applying this encoding to all valid pixels of the image, a DNTPDS feature map is obtained. The final descriptor is then built by dividing the feature map into non-overlapping blocks and concatenating the local histograms extracted from these blocks. The main advantage of DNTPDS is that it explores a larger neighborhood than standard LBP while avoiding the use of all 25 pixels of the 5×5 patch. By selecting only 16 directional pixels, it captures local variations along important orientations and radii while maintaining a compact binary encoding. This makes DNTPDS suitable for representing directional local structures and for reducing redundant information compared with descriptors that use all pixels in the neighborhood.

### 3.6. Multi-Directional Guided Mixed Mask (MDGMM-LTP)

The Multi-Directional Guided Mixed Mask-based Local Ternary Pattern (MDGMM-LTP) was introduced by El Khadiri et al. [43] as a high-performance local descriptor for texture classification. It seeks to combine the strengths of two influential families—Local Ternary Pattern (LTP) [58] and Local Directional Pattern (LDP) [59] by analysing both differential excitation and directional ordering. MDGMM-LTP starts by constructing a guided mixed mask, which is the average of four classical edge operators (Kirsch, Robinson, Prewitt and Frei–Chen) applied in eight compass directions. This hybrid kernel allows the descriptor to capture subtle texture variations across multiple orientations and spatial contexts. When evaluated on sixteen public texture datasets, MDGMM-LTP consistently outperformed a wide range of handcrafted descriptors and even deep-learning features, confirming its discriminative power and robustness.

A key innovation in MDGMM-LTP is its multi-directional guided mixed mask. Instead of relying on a single gradient operator, the method averages four directional masks—Kirsch ($KM_{i}$), Frei–Chen ($FcM_{i}$), Robinson ($RM_{i}$) and Prewitt ($PM_{i}$)—in each of the eight directions i∈{0,…,7}. The mixed mask is defined element-wise by

$$M_{f}\left(KM_{i},FcM_{i},RM_{i},PM_{i}\right)=\frac{KM_{i}+FcM_{i}+RM_{i}+PM_{i}}{4}$$

producing a balanced kernel that combines different edge characteristics. In matrix form it appears as a 3×3 stencil whose entries are the average of the four masks at corresponding positions. By fusing high-frequency Kirsch edges with smoother Frei–Chen and Prewitt gradients and Robinson’s orientation sensitivity, this blended kernel enriches the local sampling pattern and mitigates the directional bias of using a single operator.

Given a 3×3 neighbourhood SI centered at pixel $I_{c}$, the guided mixed masks $\left\{GM_{i}\right\}$ are applied in eight directions. Convolution of SI with each $GM_{i}$ yields a set of directional responses ${\left\{GeR_{p}\right\}}_{p=0}^{7}$. A second, centrally applied mask—typically a second-derivative Gaussian filter—produces the central response $GeR_{c}$. These guided responses integrate directional gradients from all four mask families and provide a richer edge description than traditional LDP, which relies on a single operator.

To increase robustness against noise and illumination variations, MDGMM-LTP employs three mean values

$$\mu =\frac{I_{c}+\sum_{p=0}^{7}I_{p}}{9},$$

$$\upsilon =\frac{GeR_{c}+\sum_{p=0}^{7}GeR_{p}}{9},$$

$$\omega =\frac{1}{MN}\sum_{m=1}^{M}\sum_{n=1}^{N}I\left(m,n\right)$$

where μ is the local average of the raw intensities, υ is the local average of the guided responses, and ω is the global mean of the image. These averages serve as reference levels for thresholding, helping the descriptor remain stable under monotonic grayscale transformations.

To encode contrast variations, MDGMM-LTP uses two ternary decision functions (with a threshold τ>0)

$$\delta_{\tau ,G_{c}}^{+}\left(x,y\right)=\left\{\begin{array}{cc} 1, & x-G_{c}\geq \tau \left(y\geq 0\right),\\ 0, & \mathrm{otherwise}, \end{array}\right.$$

$$\delta_{\tau ,G_{c}}^{-}\left(x,y\right)=\left\{\begin{array}{cc} 1, & x-G_{c}\leq \tau \left(y\leq 0\right),\\ 0, & \mathrm{otherwise}. \end{array}\right.$$

Here, $G_{c}$ denotes the central intensity $I_{c}$. The positive function $\delta^{+}$ detects significant positive differences (considering both the pixel difference $x-G_{c}$ and the sign of *y*), while $\delta^{-}$ detects significant negative differences. Differences that fall within (−τ,τ) map to zero, reducing sensitivity to minor fluctuations and noise. These functions generate two ternary streams for each pixel: an upper stream (positive responses) and a lower stream (negative responses).

To incorporate directional ordering (as in LDP [59]), MDGMM-LTP uses a logical conjunction $\Lambda (\alpha_{1},\alpha_{2})$ that equals 1 only when both inputs are 1. The descriptor forms two nine-bit codes

$$GDP_{U}=\Lambda \;\left(\delta^{+}\left(\mu ,GeR_{c}\right),\delta^{+}\left(\omega ,\upsilon \right)\right)\;2^{8}+\sum_{p=0}^{7}\delta^{+}\;\left(I_{p},GeR_{p}\right)\;2^{p},$$

$$GDP_{L}=\Lambda \;\left(\delta^{-}\left(\mu ,GeR_{c}\right),\delta^{-}\left(\omega ,\upsilon \right)\right)\;2^{8}+\sum_{p=0}^{7}\delta^{-}\;\left(I_{p},GeR_{p}\right)\;2^{p}.$$

**Figure 6:**
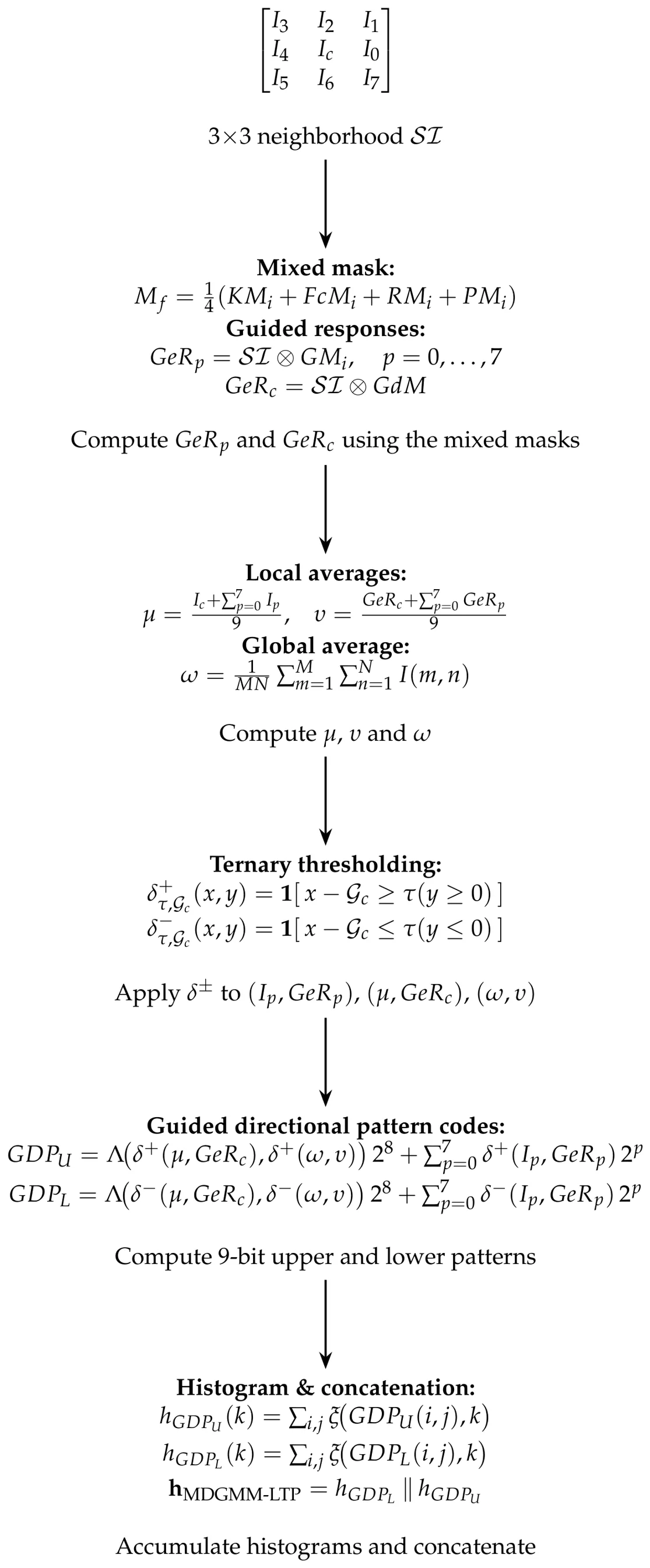
MDGMM-LTP pipeline: mixed mask construction, guided response extraction, ternary thresholding via local/global averages, guided directional pattern coding, and histogramming.

The first term in each expression encodes whether both μ and ω differences agree (positive or negative) relative to their guided responses, while the summation encodes directional dominance of the individual responses. This yields two nine-bit patterns: one for positive (upper) patterns and one for negative (lower) patterns, as illustrated in Figure 6.

Finally, histograms of $GDP_{U}$ and $GDP_{L}$ codes are concatenated to form the MDGMM-LTP feature vector

$$h_{\mathrm{MDGMM}\text{-}\mathrm{LTP}}=h_{GDP_{L}}\;∥\;h_{GDP_{U}}.$$

Concatenating these histograms yields the final 1024-bin feature vector.

### 3.7. List of Evaluated State-of-the-Art Texture Descriptors

The strong performance of LBP in many computer vision applications has motivated researchers to design more discriminative LBP-based descriptors. Its success is mainly due to its computational efficiency and its flexibility, which allow it to be adapted to a broad range of recognition tasks, including dynamic texture classification, scene analysis, medical image processing, and other non-conventional texture-related applications. Since the original work of Ojala, a large number of LBP-inspired texture descriptors have been introduced, and this family of methods continues to be actively developed. These LBP-inspired texture descriptors are mostly based on the improvement of one or more of the following original LBP elements:

1. Neighborhood topology and sampling: It is about the choice of the shape and number of neighboring pixels that should be considered to form the local feature vector.
2. Thresholding and quantization: This is to bring the resulting code down to some preset levels using thresholds.
3. Encoding and regrouping: In order to improve distinctiveness, some codes are combined to form a group of patterns.
4. Combining complementary features: Some developed methods tried to combine multiple interdependent LBP-like codes, or LBP-like with non-LBP ones.

A more detailed taxonomy of existing LBP variants is provided in [52], where the main strengths and weaknesses of these descriptors, as well as their methodological relationships, are discussed in depth. Table 1 lists the evaluated LBP-based and non-LBP descriptors in chronological order, along with their original application domains. Whenever available, the original public source code was used; otherwise, the descriptor was implemented according to the methodological details reported in the corresponding paper.

## 4. Databases Acquisition Protocol

**Figure 7:**
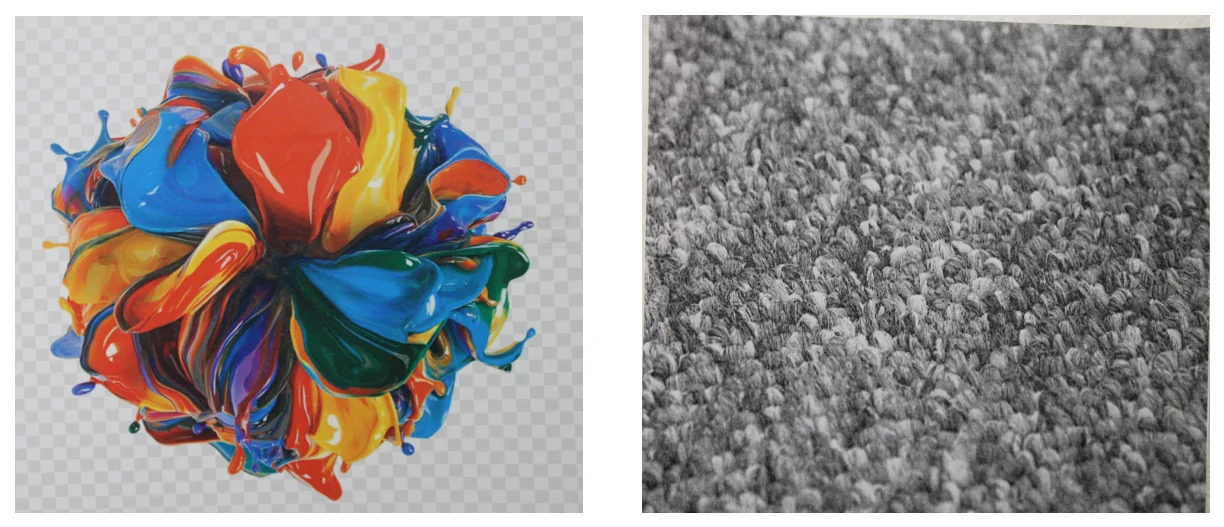
Reference patterns used during acquisition: (**left**)–color image; (**right**)–texture image.

To enable a fair, method-agnostic comparison of local feature descriptors for image-based ophthalmic lens identification, we constructed two dedicated image databases that emulate realistic viewing through ophthalmic lenses. For this purpose, two complementary categories of reference patterns were used during the image acquisition process (see Figure 7): a color-rich image and a high-frequency texture pattern. The color-based reference provides pronounced chromatic content, enabling descriptors to evaluate the influence of different optical lenses on color reproduction, light dispersion, and chromatic aberration effects. Conversely, the texture reference, characterized by fine spatial details and repetitive structural patterns, allows descriptors to detect subtle geometric degradations, including defocus, anisotropic blur, and small-scale distortions induced by variations in refractive index or surface treatments. By combining both patterns, the protocol elicits a broad spectrum of lens-induced artifacts—photometric (color-related) and geometric (structure-related)—under controlled yet representative imaging conditions.

The following subsections describe the selected ophthalmic lenses, the acquisition setup, and the construction of the two benchmark datasets.

### 4.1. Optical Lens Selection

The databases cover a representative set of ophthalmic lenses characterized by:

- **Refractive index levels:** Low (e.g., 1.50 and 1.56), medium (e.g., 1.60), and high (e.g., 1.67);
- **Anti-reflective (AR) coatings:** Standard green, blue-light blocking, and premium multilayer coatings;
- **Uniform lens geometry:** All samples used in this study are commercially available meniscus-shaped ophthalmic plano lenses with a nominal back-vertex power of 0.00 D. Here, the term *plano* refers to the nominal optical power of the lens and does not imply that both physical surfaces are flat. The anterior and posterior surfaces are designed to compensate each other optically, resulting in nominal zero corrective power. To minimize geometry-related variability, all lens categories were selected with the same nominal diameter, similar nominal thickness, identical mounting conditions, and identical image acquisition geometry. Consequently, the benchmark primarily evaluates visual differences induced by refractive index and anti-reflective coating while minimizing the influence of lens geometry.

### 4.2. Reference Patterns and Imaging Setup

For each capture, one of the two reference patterns was placed behind the lens to provide a controlled background. The imaging system comprised:

- A fixed Canon EOS 1100D digital RGB camera;
- Uniform, diffuse white LED illumination to minimize shadows and emphasize optical artifacts;
- A precision-aligned lens holder ensuring normal incidence between the camera, lens, and reference pattern.

### 4.3. Image Acquisition Process

Image acquisition was performed using a Canon EOS 1100D digital RGB camera under a fixed imaging setup. Throughout the acquisition process, the camera position, illumination conditions, lens mounting configuration, and imaging geometry were kept unchanged to ensure a fair comparison between all ophthalmic lens categories. The camera-to-lens distance was fixed at 20 cm, while the distance between the ophthalmic lens and the reference target was maintained at 18 cm.

For both reference patterns, images were acquired under the following conditions:

- Without any lens (reference class);
- Through each of the twelve commercial ophthalmic lenses, corresponding to all combinations of the four refractive indices (1.50, 1.56, 1.60, and 1.67) and the three anti-reflective coating types (green, blue, and white organic), under identical acquisition conditions.

For each class, 24 images were acquired under identical imaging conditions. The original images were subsequently cropped and resized to a resolution of 350×350 pixels prior to feature extraction.

### 4.4. Description of the Acquisition Device

**Figure 8:**
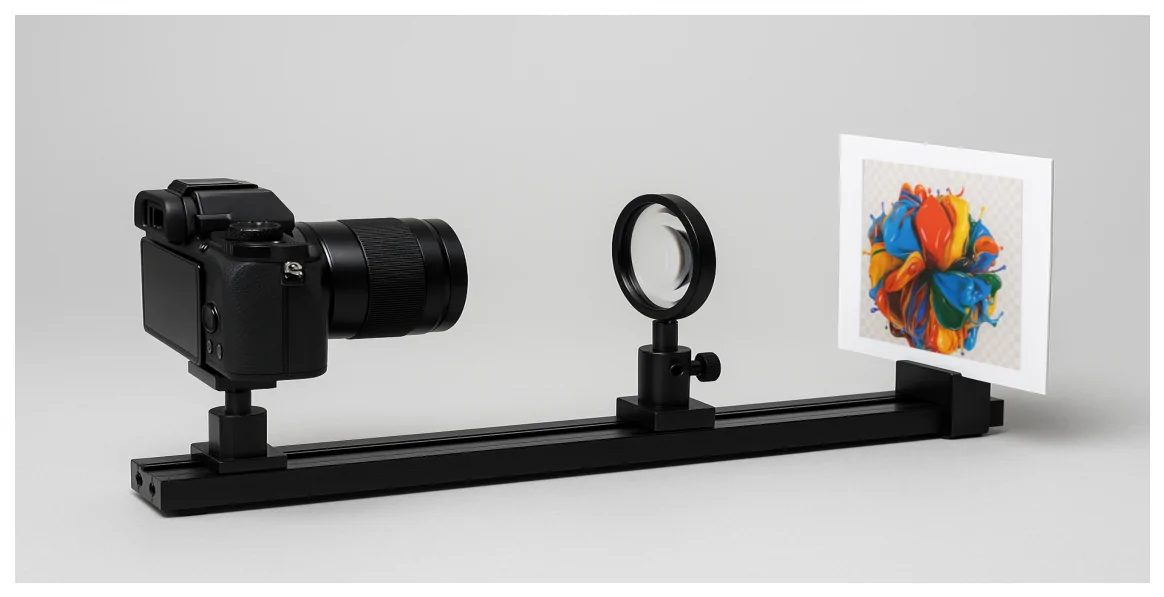
Optical acquisition device used to construct TexLensDB and ColLensDB.

The acquisition rig consists of a linear rail supporting three components aligned on a common optical axis: a Canon EOS 1100D digital RGB camera, a test lens, and a printed reference pattern. The camera is mounted on an adjustable holder to allow precise positioning. A dedicated, height-adjustable lens mount enables fine control of the lens–camera spacing. Opposite the camera, a high-contrast reference board is rigidly mounted. This configuration facilitates systematic imaging of the pattern through the lens, enabling the assessment of sharpness, geometric distortions, aberrations, and overall image quality under repeatable conditions. Figure 8 illustrates the optical acquisition device used in this work.

### 4.5. Datasets Purpose and Challenges

The resulting databases are intended to stress-test local descriptors by introducing:

- Subtle, spatially smooth distortions due to refraction and reflection;
- Minimal yet discriminative textural shifts that challenge gradient- and histogram-based encoders;
- High inter-class similarity (same reference pattern with lens-induced variations), demanding sensitivity to fine structural deformations.

Table 2 summarizes the main characteristics of the constructed datasets, namely ColLensDB and TexLensDB. Both datasets comprise 13 classes, including one reference class without a lens and twelve ophthalmic lens categories obtained from all combinations of the four most common refractive indices (1.50, 1.56, 1.60, and 1.67) and three anti-reflective coating types (green, blue, and white organic). Each ophthalmic lens category corresponds to a single commercial ophthalmic lens. Consequently, the classification task is formulated as a multi-class recognition problem.

## 5. Experimental Results

This section presents a comprehensive comparative evaluation of the proposed benchmark on the two custom-built image datasets, TexLensDB and ColLensDB. To ensure the robustness and fairness of the experimental evaluation, the same evaluation protocol was applied to all considered methods and datasets. All experiments were conducted using a split-sample validation strategy. For each dataset, three training/testing regimes were considered: (i) the standard 50% training/50% testing setting, (ii) a low-shot setting with *one* training image per class, and (iii) a moderately low-shot setting with *five* training images per class. Unless otherwise specified, the classification stage was performed using the parameter-free 1-NN classifier. The $L_{1}$ (Cityblock) distance was adopted as the default similarity measure throughout the experiments, while additional experiments were conducted using the Chi-Square and Cosine distances under the standard 50%/50% protocol. To reduce any bias related to a particular train/test partition, the classification procedure was repeated over 100 random splits, and the final recognition rates were reported as average accuracies over all runs. To ensure a fair and unbiased comparison, exactly the same training and testing splits were used for all handcrafted descriptors, distance metrics, supervised classifiers, and pretrained deep feature extractors. The following subsections provide a comprehensive experimental evaluation by analyzing descriptor performance under different training regimes, investigating the influence of the adopted distance metrics, comparing handcrafted descriptors with supervised SVM classifiers and pretrained deep feature extractors, assessing the statistical significance of the obtained results, and finally reporting the implementation details required to ensure reproducibility.

### 5.1. Comparative Assessment of Performance

#### 5.1.1. Experiment #1: Performance Stability Across Both Datasets

Table 3 presents the average recognition rates obtained by the evaluated state-of-the-art texture descriptors on TexLensDB and ColLensDB under the standard 50%/50% training/testing protocol. The reported results provide a comprehensive comparison of the evaluated descriptors and enable an assessment of both their discriminative capability and their robustness across the different similarity measures considered in this study.

Overall, the experimental results indicate that several recent LBP-based and LBP-inspired descriptors consistently achieve high recognition rates across the three evaluated distance metrics. On TexLensDB, descriptors such as RGP, DNTPDS, RCSP, MDGMMLTP, ARLBP, QS-LBP, FDLBP, PGMO-MSTP, RFD and OD-LBP consistently rank among the most competitive methods. Notably, when combined with the Chi-Square distance, RGP achieves one of the highest recognition rates on this dataset, although the relative ranking of the leading descriptors varies slightly depending on the adopted similarity measure. In particular, the Chi-Square distance further improves the performance of several histogram-based descriptors, leading to the highest recognition rates on this dataset, whereas the L1 distance remains highly competitive for most of the top-performing methods. These results demonstrate that advanced local encoding strategies exploiting enlarged neighborhoods, directional relationships, radial or angular transitions, and enriched local sampling effectively capture the subtle micro-textural variations present in TexLensDB. These observations indicate that the discriminative power of the best-performing descriptors primarily originates from the quality of their local representations, while the choice of the similarity measure plays only a secondary role.

For ColLensDB, RGP stands out as the best-performing descriptor, achieving the highest recognition rate when combined with the Chi-Square distance. RCSP, MDGMMLTP, FDLBP, DNTPDS, PGMO-MSTP, and QS-LBP also rank among the top-performing descriptors across the three evaluated distance metrics. These results suggest that ColLensDB benefits from descriptors capable of jointly encoding local texture structures, spatial relationships, and appearance variations, thereby providing a richer representation of chromatic and structural information. In general, the Chi-Square distance tends to provide the highest recognition rates on this dataset, although the improvements over the L1 distance remain relatively moderate for the strongest descriptors.

A comparison of the three distance metrics reveals that the influence of the distance measure depends on the descriptor under consideration. For most of the evaluated descriptors, the Chi-Square distance provides the highest recognition rates, which is expected since it is specifically designed for comparing histogram distributions. In contrast, the L1 distance remains highly competitive and often produces results very close to those obtained with the Chi-Square metric. The Cosine distance generally leads to slightly lower recognition rates for most descriptors, although several methods, including DNTPDS, RCSP, QS-LBP, MDGMMLTP, and OD-LBP, maintain strong performance regardless of the selected metric. These observations demonstrate the robustness of the best-performing descriptors with respect to the choice of the distance measure. Moreover, the relatively small performance differences observed between the L1 and Chi-Square distances indicate that descriptor design has a greater influence on recognition performance than the choice of the similarity measure itself.

Some descriptors also exhibit a pronounced dataset dependency independently of the adopted distance metric. For example, SWOBP consistently achieves much higher recognition rates on ColLensDB than on TexLensDB, while MCLBP also performs substantially better on the color-based dataset. Such a consistent trend across the three evaluated distance metrics can be attributed to their color-oriented encoding mechanisms, which are naturally better suited to ColLensDB. Conversely, descriptors specifically designed to characterize fine local intensity variations generally achieve superior performance on TexLensDB, where the discriminative information is mainly conveyed by grayscale micro-textures. These observations further confirm that descriptor effectiveness depends not only on the local encoding strategy but also on the nature of the visual information contained in the dataset.

Taken together, these results show that RGP, DNTPDS, RCSP, MDGMMLTP, ARLBP, QS-LBP, FDLBP, PGMO-MSTP, RFD and OD-LBP remain among the most competitive methods, irrespective of the adopted similarity measure. Among these descriptors, RGP exhibits the most consistent overall performance across both datasets, particularly when combined with the Chi-Square distance. These findings further highlight the effectiveness of advanced local encoding strategies exploiting enriched neighborhood sampling, directional relationships, radial and angular interactions, and robust thresholding mechanisms for constructing highly discriminative representations of ophthalmic lens optical characteristics. Overall, these findings demonstrate that carefully designed handcrafted texture descriptors remain highly effective for image-based ophthalmic lens identification and constitute a strong baseline for future learning-based approaches.

#### 5.1.2. Experiment #2: Performance Stability as a Function of Training Set Size

This experiment investigates how the performance of the evaluated state-of-the-art descriptors is affected by the size of the training set. The objective is to assess their stability and robustness under limited-data conditions, which is particularly important for image-based identification of ophthalmic lens characteristics, where only a small number of samples may be available for certain lens categories. Table 4 summarizes the classification accuracies obtained on TexLensDB and ColLensDB when the number of training images per class is limited to N = 1 and N = 5. The table also reports the absolute accuracy gain between the two training regimes, the mean standard deviation (Mean Std), and the Global Average Performance (GAP), computed over the four reported accuracy values for each descriptor.

As discussed in the previous subsection, the Chi-Square distance generally provides the highest recognition rates under the standard 50%/50% training/testing protocol. However, the purpose of the present experiment is to investigate the influence of the training set size independently of the adopted similarity measure. Therefore, to maintain consistency with the original benchmarking protocol and to facilitate direct comparisons with the previously reported low-shot results, the stability analysis presented in this section is carried out using the conventional L1 distance.

When only one training image per class is used, a clear performance gap is observed among the evaluated descriptors. CSQP achieves the best overall one-shot performance, with 83.18% on TexLensDB and 82.37% on ColLensDB. It is followed by LOOP, which reaches 85.71% on TexLensDB and 73.81% on ColLensDB, and by DNTPDS, which obtains 81.46% and 72.10%, respectively. Other competitive descriptors include RGP, ARLBP, RCSP, FDLBP, FLNIP, MDGMMLTP, and QS-LBP. The conclusions drawn from the analysis of these results are consistent with those previously reported in Section 5.1.1. In particular, descriptors based on structured neighborhood relationships, directional sampling, compact quantization, and enriched local encoding generally show greater robustness when only a very limited number of training samples is available.

Increasing the number of training images from N=1 to N=5 leads to a substantial improvement for almost all descriptors. This confirms that the evaluated handcrafted methods benefit significantly from a more representative training set. In the five-shot setting, CSQP again provides the strongest average performance across the two datasets, with 95.98% on TexLensDB and 91.76% on ColLensDB. RCSP also performs very well, reaching 95.31% and 92.09%, followed by FDLBP with 95.53% and 91.50%. DNTPDS, MDGMMLTP, ARLBP, RGP, and QS-LBP also remain among the most competitive methods, confirming their effectiveness in moderate low-shot conditions.

The absolute gain from N=1 to N=5 is highly descriptor-dependent. Some methods exhibit very large improvements, such as LQCH, PLGF, RTLNP, LGONBP, SBP, WMLBP, and ARCSLBPrn. For example, LQCH improves by +31.19 percentage points on TexLensDB and +24.78 percentage points on ColLensDB, while PLGF improves by +27.48 and +24.94 percentage points, respectively. These large gains suggest that such descriptors are strongly dependent on the availability of additional training samples and may be less stable in extremely low-shot conditions.

In contrast, descriptors such as CSQP, LOOP, DNTPDS, ARLBP, RCSP, FDLBP, and RGP show more moderate gains. For instance, CSQP exhibits gains of +12.80 percentage points on TexLensDB and +9.39 percentage points on ColLensDB, while LOOP shows gains of +10.86 and +10.26 percentage points, respectively. DNTPDS also shows moderate gains of +14.82 and +18.54 percentage points. This behavior suggests that these descriptors are able to extract relatively stable and discriminative features even when the training set is very limited.

The GAP values provide a global view of the average performance achieved by each descriptor across both datasets and both training regimes. From this perspective, CSQP obtains the highest GAP with 88.32%, followed by DNTPDS (85.12%), LOOP (85.04%), RCSP (84.19%), ARLBP (83.89%), RGP (83.84%), FDLBP (83.62%), and MDGMMLTP (83.31%). These results show that the most competitive descriptors are those that maintain high accuracy in both one-shot and five-shot configurations, rather than those that only exhibit large gains when more training samples are added.

The Mean Std values provide complementary information about stability across random train/test partitions. Lower values indicate that the descriptor is less sensitive to the specific choice of training and testing samples. CSQP achieves the lowest Mean Std (2.97), followed by LOOP (3.18), DNTPDS (3.28), ARLBP (3.34), FDLBP (3.35), FLNIP (3.36), RCSP (3.40), and QS-LBP (3.44). These descriptors therefore show a more stable behavior under repeated random splits. However, stability should be interpreted together with accuracy. For example, CSQP combines both the lowest Mean Std and the highest GAP, whereas other descriptors may show acceptable stability but lower recognition rates.

Overall, the results show that the size of the training set has a significant impact on the recognition performance of handcrafted texture descriptors. Most methods improve substantially when moving from one-shot to five-shot classification, but the magnitude of the gain varies from one descriptor to another. Descriptors such as CSQP, DNTPDS, LOOP, RCSP, ARLBP, FDLBP, RGP, MDGMMLTP, and QS-LBP provide the best compromise between accuracy, stability, and robustness under limited-data conditions. These findings confirm that enriched local texture descriptors remain highly relevant for image-based identification of ophthalmic lens characteristics, especially when the available training data are limited.

#### 5.1.3. Experiment #3: Comparison with Support Vector Machine Classifiers

Although the parameter-free 1-NN classifier has been adopted throughout the previous experiments because of its simplicity and effectiveness for histogram-based texture descriptors, it is also important to investigate whether a more sophisticated supervised classifier can further improve the recognition performance. Support Vector Machines (SVMs) are among the most widely used classifiers in computer vision and texture analysis due to their strong generalization capability. However, their performance strongly depends on the choice of the kernel function and the associated hyperparameters, particularly the penalty parameter C and the kernel parameter γ. As in many texture classification problems, different values of C and γ have been reported in the literature [96,97,98,99,100]. In this work, the parameter values recommended in [100] were adopted because preliminary experiments showed that they provided the best overall performance on the considered datasets. To keep the comparison focused and computationally tractable, the SVM experiments were conducted only on the ten top-ranked texture descriptors identified in the previous comparative evaluation. Table 5 compares the recognition rates obtained with the reference 1-NN classifier (L1 distance) and three SVM classifiers employing Linear, Polynomial, and RBF kernels.

Overall, the SVM classifiers provide competitive recognition rates on both TexLensDB and ColLensDB. However, the reference 1-NN classifier consistently outperforms the evaluated SVM classifiers for the majority of the considered descriptors. Only a limited number of descriptors, such as PGMO-MSTP and RGP on TexLensDB, show a slight improvement when classified with SVM, whereas for most descriptors the recognition rates remain lower than those obtained with the reference 1-NN classifier. These results indicate that the evaluated texture descriptors already produce highly discriminative feature representations, allowing a simple nearest-neighbor classifier to separate the different ophthalmic lens categories effectively.

A comparison among the three SVM kernels further reveals that only small performance differences are observed between the Linear, Polynomial, and RBF kernels. Although the Polynomial kernel occasionally achieves the highest recognition rates on TexLensDB, no kernel consistently dominates the others across both datasets. This observation suggests that, for the proposed application, the discriminative capability of the extracted texture descriptors has a greater impact on the final recognition performance than the particular choice of the SVM kernel. Taken together, these results indicate that the discriminative capability of well-designed handcrafted texture descriptors plays a more important role than classifier complexity for accurate image-based identification of ophthalmic lens optical characteristics. Consequently, employing a more sophisticated classifier does not necessarily translate into higher recognition accuracy.

#### 5.1.4. Experiment #4: Statistical Significance of Accuracy Improvements

**Figure 9:**
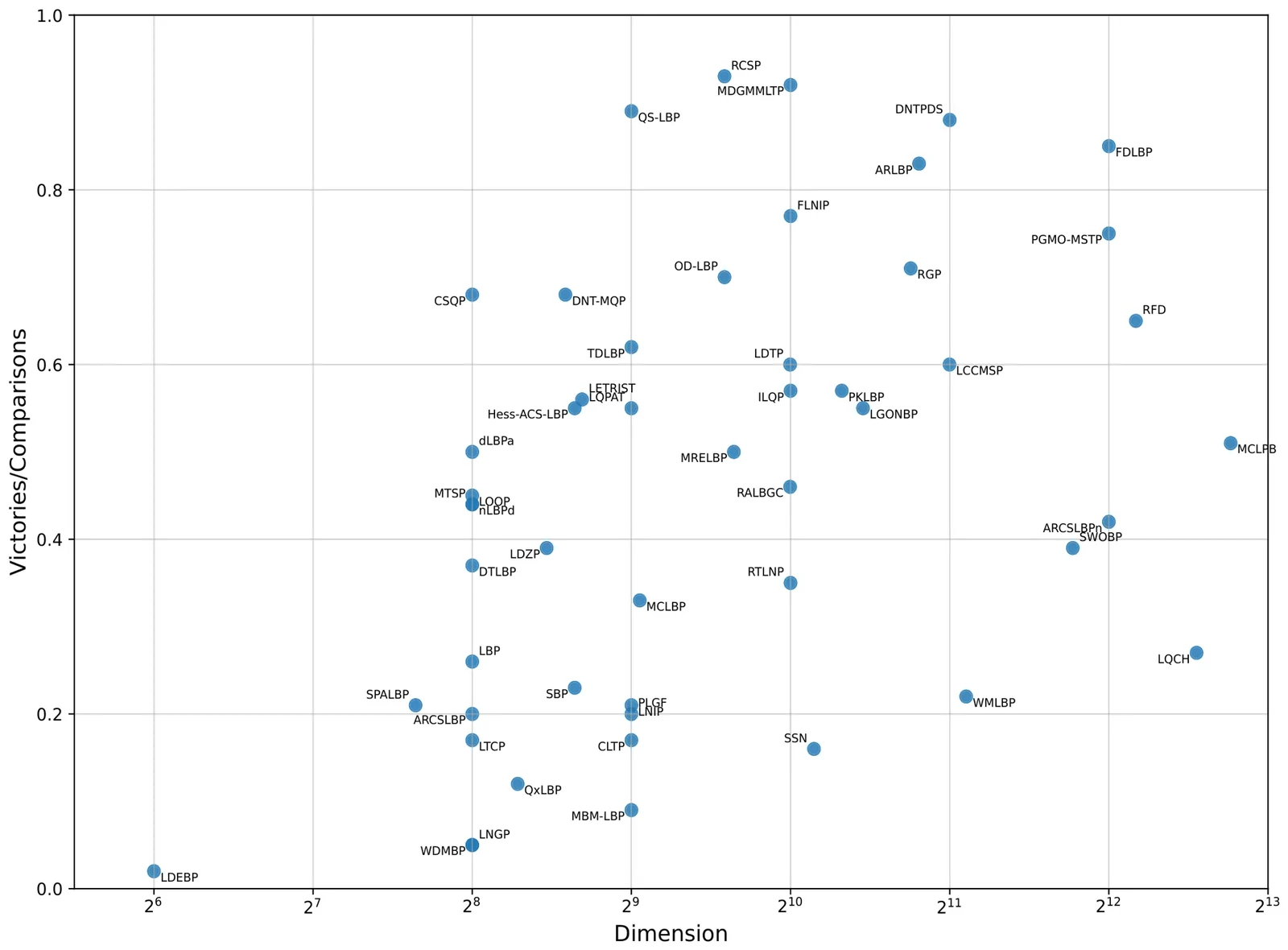
Ranking results on the two used datasets.

In addition to the accuracy-based evaluation, a statistical analysis was conducted to examine whether the observed differences between descriptors are consistent across the experimental runs. To this end, we adopted the ranking strategy based on the Wilcoxon signed-rank test, as used in [51,71]. Since all descriptors were evaluated under identical random train/test partitions, the comparisons were performed in a paired setting, allowing a fair assessment of the relative behavior of each method with respect to the others. Table 6 shows the reached ranking results according to the normalized number of victories (number of wins/(number of used databases × (number of evaluated methods − 1))) realized by each descriptor on the two considered databases. The “Victories/comparisons” column reports the proportion of pairwise comparisons in which a given descriptor achieves a statistically significant advantage over its competitors. Thus, larger values indicate a higher ability to consistently outperform other methods across the repeated experimental trials. The last column gives the dimensionality of the feature vector associated with each descriptor. Figure 9 illustrates the produced classification results in the form of a scatter plot. The X-axis is the dimension of feature vectors, while the Y-axis is the normalized number of victories reached by each evaluated texture operator.

The results show that RCSP achieves the best statistical ranking, with a victory ratio of 0.93. This indicates that RCSP obtains statistically significant wins against most of the evaluated descriptors. MDGMMLTP ranks second with a score of 0.92, followed by QS-LBP (0.89), DNTPDS (0.88), FDLBP (0.85), and ARLBP (0.83). These results identify this group of descriptors as the most reliable methods from a statistical point of view. A closer look at the ranking reveals that the strongest methods are generally recent LBP-inspired descriptors that exploit richer local structures. In particular, RCSP achieves the highest score by combining radial-cross and symmetric neighborhood relationships, while MDGMMLTP ranks second, confirming the relevance of multi-directional guided mixed-mask ternary encoding for this task. DNTPDS benefits from directional sampling, ARLBP captures angular and radial transitions, and QS-LBP uses an extended star-like neighborhood. Similarly, FDLBP, PGMO-MSTP, RGP, OD-LBP, DNT-MQP, and CSQP obtain relatively high scores, confirming the importance of advanced neighborhood modeling and discriminative local encoding for capturing subtle lens-induced visual variations.

By contrast, several simpler or more conventional descriptors appear in the lower part of the ranking. The standard LBP descriptor obtains a score of only 0.26, while methods such as SBP, WMLBP, SPALBP, LNIP, ARCSLBP, PLGF, CLTP, LTCP, SSN, QxLBP, MBM-LBP, LNGP, WDMBP, and LDEBP achieve lower victory ratios. This suggests that basic binary thresholding or limited local sampling may not be sufficient to represent the weak texture and appearance changes induced by ophthalmic lenses.

The feature dimensionality also provides useful insight into the balance between compactness and discriminative power. Some top-ranked descriptors, such as RCSP and QS-LBP, achieve strong statistical performance with moderate feature sizes of 768 and 512, respectively. In contrast, other competitive methods rely on larger representations, such as FDLBP and PGMO-MSTP with 4096 features, RFD with 4608 features, and MCLPB with 6962 features. Therefore, the ranking does not depend only on dimensionality; rather, it reflects the ability of each descriptor to encode relevant local structures for the considered classification task.

Overall, the Wilcoxon-based analysis supports the conclusions obtained from the recognition-rate evaluation. The most statistically reliable descriptors are those that combine enriched neighborhood sampling, directional or symmetric relationships, and discriminative local encoding. In particular, RCSP, MDGMMLTP, QS-LBP, DNTPDS, FDLBP, and ARLBP provide the strongest statistical evidence of effectiveness for image-based identification of ophthalmic lens characteristics.

#### 5.1.5. Experiment #5: Comparison with Pretrained Deep Feature Extractors

To further position the proposed benchmark with respect to modern visual representations, additional experiments were conducted using representative pretrained convolutional neural networks (CNNs) and Vision Transformers (ViTs). It should be emphasized that these pretrained models were used exclusively as fixed feature extractors rather than as end-to-end classifiers. Their output embeddings were therefore treated as feature representations and evaluated under exactly the same experimental protocol adopted for handcrafted descriptors, namely identical 100 random 50%/50% train–test splits and the same parameter-free 1-NN classifier. Consequently, the comparison focuses exclusively on the discriminative capability of the extracted feature representations rather than on differences arising from the classifier itself.

Unlike handcrafted texture descriptors, which generally produce histogram-based representations naturally compared using histogram distances such as the Chi-Square metric, pretrained CNN and Transformer models generate continuous feature embeddings. Consequently, only the L1 and Cosine distances were considered for deep features, whereas the Chi-Square distance was not retained because it is not appropriate for continuous embedding vectors. Preliminary experiments showed that the L1 and Cosine distances yielded nearly identical recognition rates for all evaluated deep models. Therefore, for the sake of clarity, only the L1 results are reported. Table 7 summarizes the best recognition rates obtained by the top-performing handcrafted texture descriptors and the representative pretrained deep feature extractors.

The results show that pretrained deep feature extractors achieve excellent recognition performance on both datasets. In particular, EfficientNet-B0 achieves the highest recognition rate on TexLensDB (99.38%), whereas MobileNetV3-Large obtains the best performance on ColLensDB (99.63%). ResNet18, ViT-B32, and Swin-T also achieve consistently high recognition rates, confirming the effectiveness of pretrained deep representations for ophthalmic lens image analysis.

Despite these strong results, the best handcrafted texture descriptors remain highly competitive. On TexLensDB, several handcrafted descriptors achieve recognition rates above 98%, with RGP and QS-LBP reaching 98.57% and 98.66%, respectively. Similarly, on ColLensDB, RGP attains 98.71%, remaining close to several pretrained deep models. These observations indicate that carefully designed handcrafted descriptors are still capable of capturing highly discriminative texture characteristics without requiring large-scale network training or fine-tuning.

Overall, the experimental results demonstrate that both handcrafted and pretrained deep representations provide highly effective solutions for ophthalmic lens identification. While pretrained deep models generally achieve the highest recognition rates, the performance gap with the best handcrafted descriptors remains relatively small, confirming that handcrafted texture analysis continues to constitute a competitive alternative for this application. Moreover, unlike pretrained deep models, handcrafted texture descriptors do not require large-scale external datasets, GPU-based training, or transfer learning, making them particularly attractive for small-scale industrial inspection problems where annotated data are limited.

### 5.2. Implementation, Execution and Reproducible Research

The evaluated methods were implemented in MATLAB^®^ R2018b. All experiments were performed on a laptop featuring an Intel© Core^™^ i7-11800H CPU @ 2.30 GHz (Intel, Santa Clara, CA, USA) with 8 cores, 16 GB RAM, and an NVIDIA GeForce RTX 3070 GPU (Nvidia, Santa Clara, CA, USA), running the 64-bit Windows 11 operating system. To ensure research reproducibility, all resources required to replicate the experiments—including source codes, images, and the corresponding training/validation splits—are available from the corresponding author upon request.

**Figure 10:**
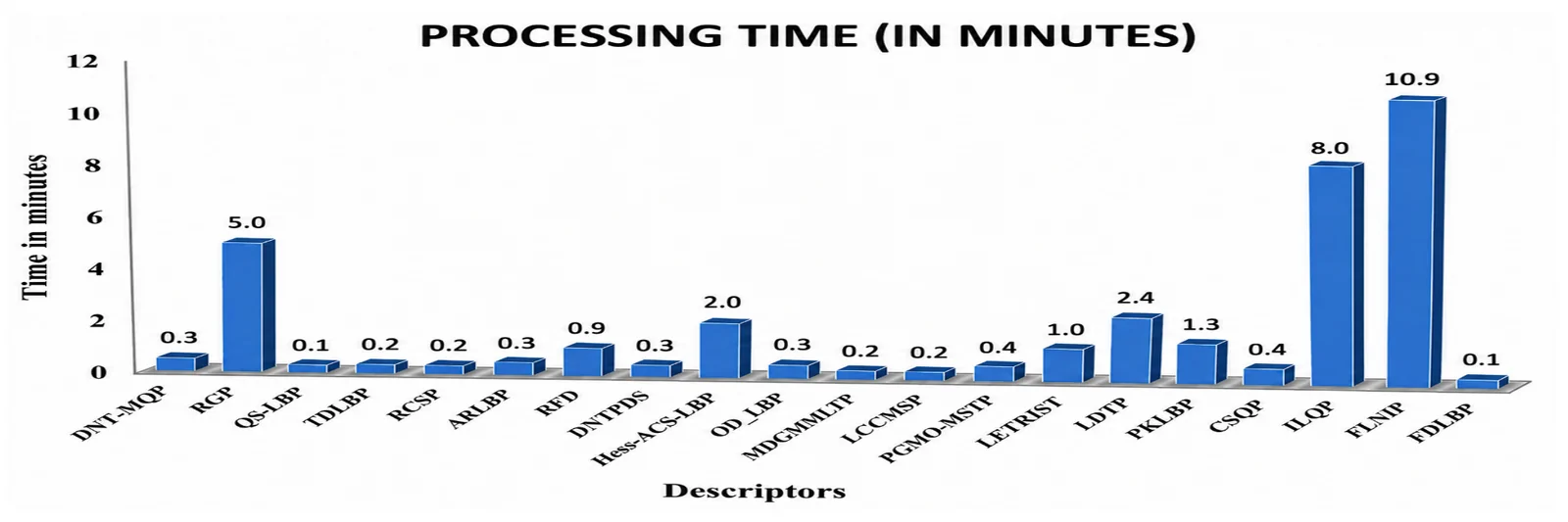
Comparison of the processing times of the evaluated texture descriptors. The reported execution times, expressed in minutes, include feature extraction, distance computation, and 1-NN classification on both optical lens datasets.

The computational cost of each evaluated descriptor was measured on the two optical lens datasets used in this study, comprising a total of 13×24×2=624 images. The reported execution time includes the complete recognition pipeline, namely feature extraction, distance computation, and 1-NN classification. Figure 10 summarizes the processing time, expressed in minutes, for the 20 top-ranked texture descriptors according to the Wilcoxon-based ranking presented in Table 6.

As illustrated in Figure 10, the computational requirements vary considerably from one descriptor to another. Most descriptors complete the entire recognition process in less than one minute, including QS-LBP and FDLBP (0.1 min), TDLBP, RCSP, MDGMMLTP, and LCCMSP (0.2 min), as well as DNT-MQP, ARLBP, DNTPDS, and OD-LBP (0.3 min). Other competitive descriptors, such as PGMO-MSTP and CSQP, also remain computationally efficient, requiring only 0.4 min, whereas RFD, LETRIST, Hess-ACS-LBP, PKLBP, and LDTP require between 0.9 and 2.4 min. In contrast, ILQP and FLNIP are the most computationally demanding methods, requiring approximately 8 and 10.9 min, respectively, while RGP requires about 5 min. These results indicate that several of the best-performing descriptors achieve excellent recognition accuracy while maintaining a relatively low computational cost, making them attractive candidates for practical non-destructive ophthalmic lens inspection systems.

From a practical viewpoint, these results reveal a favorable trade-off between computational efficiency and recognition performance. In particular, descriptors such as MDGMMLTP, DNTPDS, RCSP, DNT-MQP, QS-LBP, and ARLBP combine high recognition rates with short execution times, whereas some more computationally intensive methods do not necessarily provide proportional gains in classification performance. This observation suggests that carefully designed local texture representations can simultaneously offer high discriminative capability and computational efficiency, making them suitable for real-time or large-scale optical lens inspection applications.

## 6. Conclusions

This paper addressed the image-based identification of ophthalmic lens characteristics, with particular emphasis on refractive index and anti-reflective coating type. This problem was formulated as a non-destructive optical inspection task, where the visual effects induced by ophthalmic lenses are interpreted as subtle texture and appearance variations produced by the interaction between the lens material and a controlled visual target. To the best of our knowledge, this is one of the first systematic benchmark studies devoted to the use of local texture descriptors for the image-based identification of ophthalmic lens characteristics. To support this investigation, two dedicated ophthalmic lens image datasets, namely TexLensDB and ColLensDB, were constructed under controlled acquisition conditions using consistent visual targets.

A comprehensive experimental benchmark was conducted using a large set of state-of-the-art handcrafted local texture descriptors, with particular focus on LBP-like methods. The evaluated descriptors cover different methodological strategies, including dense local encoding, extended neighborhood sampling, directional and radial relationships, ternary or binary thresholding, filter-based representations, color texture modeling, and graph- or structure-inspired approaches. In addition, representative pretrained deep feature extractors, including convolutional neural networks (CNNs) and Vision Transformers (ViTs) were evaluated under exactly the same experimental protocol, allowing a fair comparison between handcrafted and deep feature representations. The experimental results demonstrate that both handcrafted texture descriptors and pretrained deep feature extractors provide highly discriminative representations for ophthalmic lens identification. Although pretrained deep models generally achieve the highest recognition rates, several handcrafted descriptors remain highly competitive while offering lower computational complexity and requiring neither network training nor fine-tuning. These findings confirm that handcrafted texture analysis continues to constitute an effective and attractive solution for image-based ophthalmic lens inspection, particularly in applications involving limited annotated data.

Future work will focus on several directions. First, the datasets will be extended to include a wider range of ophthalmic lens materials, refractive indices, coatings, and optical powers. Second, more realistic acquisition conditions will be investigated, including uncontrolled illumination, specular reflections, lens tilt, defocus, surface contamination (e.g., dust particles and micro-scratches), and positioning tolerances in automated inspection systems. Third, geometric and photometric correction strategies may be integrated to improve robustness in practical inspection scenarios. In addition, the trade-off between feature dimensionality, recognition accuracy, and real-time computational efficiency deserves further investigation for high-throughput industrial inspection systems. Finally, hybrid handcrafted–deep learning approaches, lightweight fine-tuned deep models, and domain-adaptation techniques will be explored to further improve the identification of ophthalmic lens characteristics under limited-data and real-world conditions.

## Figures and Tables

**Table 1:** Summary of state-of-the-art texture descriptors studied in this paper.

$N^{\circ }$	Complete Name	Acronym	Year	Ref	Application
1	Local Binary Patterns	LBP	1996	[24]	Texture classification
2	Directional LBP	${\mathrm{dLBP}}_{\alpha }$	2015	[60]	Texture classification
3	Local Binary Patterns by Neighborhoods	nLBP_d	2015	[60]	Texture classification
4	Median Robust Extended LBP	MRELBP	2015	[61]	Texture classification
5	Multi-scale co-occurrence local binary pattern	MCLPB	2015	[62]	Texture classification
6	Local Quantization Code Histogram	LQCH	2016	[63]	Texture classification
7	Statistical Binary Patterns	SBP	2016	[64]	Texture classification
8	Local Quadruple Pattern	LQPAT	2017	[65]	Face recognition
9	Centre Symmetric Quadruple Pattern	CSQP	2018	[66]	Face recognition
10	Local Optimal-Oriented Pattern	LOOP	2018	[67]	Lepidoptera species recognition
11	Repulsive-and-Attractive LBGC	RALBGC	2018	[68]	Texture classification
12	Locally Encoded Transform Feature Histogram	LETRIST	2018	[69]	Motion texture analysis
13	Local neighborhood intensity pattern	LNIP	2018	[70]	Texture classification
14	Local Concave-and-Convex Micro-Structure Patterns	LCCMSP	2018	[71]	Texture classification
15	Local Directional ZigZag Pattern	LDZP	2018	[72]	Texture classification
16	Local Directional Ternary Pattern	LDTP	2018	[73]	Texture classification
17	Pattern of Local Gravitational Force	PLGF	2019	[74]	Face recognition and Texture classification
18	R-Theta Local Neighborhood Pattern	RTLNP	2019	[75]	Face recognition
19	Improved Local Quinary Patterns	ILQP	2019	[76]	Texture classification
20	Attractive-and-Repulsive Center-Symmetric LBP	ARCSLBP	2019	[77]	Texture classification
21	Frequency Decoded Local Binary Pattern	FDLBP	2019	[78]	Face Retrieval
22	Spatio-Spectral Networks	SSN	2020	[79]	Color-Texture Analysis
23	Spatially Weighted Order Binary Pattern	SWOBP	2020	[80]	Color texture classification
24	Fractional Local Neighborhood Intensity Pattern	FLNIP	2020	[81]	Texture classification
25	Multi-Resolution Intrinsic Texture Geometry LBP	Hess-ACS-LBP	2020	[82]	Texture classification
26	Directional Thresholded LBP	DTLBP	2020	[83]	Texture classification
27	Pyramid and Multi-Kernel Based LBP	PKLBP	2020	[84]	Texture recognition
28	Local Grouped Order Pattern and Non-local B-Pattern	LGONBP	2020	[41]	Texture classification
29	ARCSLBP (rn)	ARCSLBPrn	2020	[85]	Texture classification
30	Directional Neighborhood Topologies	DNT-MQP	2020	[86]	Texture classification
31	Local Directional Edge Binary Patterns	LDEBP	2020	[87]	Biomedical image retrieval
32	Orthogonal Difference LBP	OD-LBP	2021	[34]	Face Recognition
33	Quaternionic Extended LBP	QxLBP	2021	[88]	Color texture classification
34	Radial Cross Symmetric Pattern	RCSP	2021	[56]	Facial expression recognition
35	Wavelet Domain Majority Coupled Binary Pattern	WDMBP	2021	[89]	Texture classification
36	Multiple Channels LBP	MCLBP	2021	[90]	Color texture classification
37	Petersen Graph Multi-Orientation Multi-Scale T-Pattern	PGMO-MSTP	2021	[42]	Texture classification
38	Circumferential Local Ternary Pattern	CLTP	2022	[37]	Anti-counterfeiting pattern identification
39	Local Neighborhood Gradient Pattern	LNGP	2022	[91]	Image retrieval
40	LTP based Multi-Directional Guided Mixed Mask	MDGMM-LTP	2022	[43]	Texture classification
41	Dual Neighborhood Thresholding Patterns	DNTPDS	2023	[57]	Face recognition
42	Multi-Scale Ternary and Septenary Patterns	MTSP	2023	[92]	Texture classification
43	Local Triangular Coded Pattern	LTCP	2023	[93]	Texture classification
44	Scale and Pattern Adaptive LBP	SPALBP	2024	[94]	Texture classification
45	T-Region and Diagonal Elements LBP	TDLBP	2024	[31]	Finger-biometric classification
46	Robust Face Descriptor	RFD	2024	[95]	Face recognition
47	Wavelet Domain Multi-Scale LBP	WMLBP	2025	[49]	Texture classification
48	Multi-Block and Mean-Based LBP	MBM-LBP	2025	[28]	Facial image retrieval
49	Regional Gradient Pattern	RGP	2025	[48]	Texture classification
50	Quad Star LBP	QS-LBP	2025	[27]	Breast cancer recognition
51	Angular and Radial LBP	ARLBP	2025	[26]	X-ray pneumonia detection

**Table 2:** Summary of the constructed ophthalmic lens image datasets.

No.	Name	Classes	Samples per Class	Sample Resolution (Pixels)	Image Format	Predefined Train/Test Sets?
1	TexLensDB	13	24	350×350	color (png)	yes (100 splits)
2	ColLensDB	13	24	350×350	color (png)	yes (100 splits)

**Table 3:** Recognition accuracy (%) obtained using three distance metrics with the 1-NN classifier. The best result for each dataset and descriptor is highlighted in bold.

Texture Descriptor	L1		Chi-Square		Cosine	
	TexLensDB	ColLensDB	TexLensDB	ColLensDB	TexLensDB	ColLensDB
DNT-MQP	97.66	94.93	97.87	95.49	97.22	90.20
RALBGC	96.75	93.18	95.91	93.40	96.32	89.18
ARCSLBP	90.38	87.82	94.16	84.46	87.09	87.26
ARCSLBPn	96.69	91.91	97.60	90.77	90.37	85.88
RGP	96.45	97.29	**98.57**	**98.71**	88.95	94.31
WMLBP	95.42	84.73	96.03	91.17	94.54	80.88
QS-LBP	**98.24**	96.07	**98.66**	95.65	**98.00**	95.19
MBM-LBP	87.96	80.86	94.63	91.46	82.18	77.48
TDLBP	97.52	93.80	96.19	91.97	96.91	91.26
SPALBP	90.44	88.16	94.08	89.07	88.88	87.37
MTSP	96.71	93.33	97.18	93.55	95.60	92.41
LNGP	86.93	66.12	84.61	60.89	84.72	62.05
CLTP	93.65	82.97	96.81	86.73	89.41	72.85
WDMBP	82.15	67.52	79.54	56.45	81.41	65.59
RCSP	**98.30**	96.59	**98.52**	95.74	97.78	96.19
ARLBP	**98.33**	95.16	97.73	90.57	97.61	93.65
RFD	**98.05**	92.63	**98.40**	91.24	87.53	81.21
DNTPDS	**98.42**	95.66	**98.37**	94.90	**98.45**	96.21
Hess-ACS-LBP	97.59	93.08	**98.08**	96.40	96.52	91.35
OD-LBP	97.86	94.61	**98.05**	93.54	97.55	93.88
MDGMMLTP	**98.20**	96.76	97.25	94.01	97.75	94.60
LGONBP	93.57	95.89	95.24	96.75	89.62	94.62
LCCMSP	97.06	93.97	97.08	93.12	94.16	89.44
LBP	95.69	86.21	94.78	81.27	94.06	86.80
PGMO-MSTP	97.08	96.22	97.75	95.14	96.32	94.25
SBP2	94.42	86.55	95.36	90.82	92.12	74.06
LETRIST	97.61	93.32	97.72	94.30	95.20	90.32
PLGF	94.39	84.63	94.57	83.09	94.34	82.04
LDTP	97.28	94.15	96.70	91.89	96.80	93.84
nLBPd	96.51	93.14	95.84	93.99	96.60	91.46
LOOP	97.55	87.54	96.41	85.06	97.72	86.10
LQCH	92.65	90.54	91.60	86.57	91.64	87.29
PKLBP	97.63	93.19	96.63	93.01	97.41	91.60
CSQP	97.72	94.63	96.37	92.68	97.20	94.42
QxLBP	88.25	84.94	93.77	83.12	80.22	79.02
SWOBP	66.96	95.38	67.31	96.05	57.73	90.30
MCLBP	86.75	93.85	86.56	94.60	82.86	92.06
SSN	79.61	89.52	79.23	46.86	65.24	84.76
LQPAT	96.64	94.87	96.67	92.48	95.93	89.82
LDEBP	81.43	60.43	82.75	59.09	82.92	60.84
ILQP	96.66	95.06	96.54	94.26	95.57	93.07
DTLBP	96.49	91.09	95.49	89.30	95.84	89.93
MRELBP	95.10	94.75	96.98	94.02	89.86	91.15
dLBPa	96.42	94.22	96.42	91.88	95.55	92.32
LDZP	96.65	91.74	97.05	89.45	94.75	84.74
MCLPB	97.67	91.42	97.87	88.12	93.16	92.54
LNIP	95.63	82.26	94.17	75.68	95.88	82.81
FLNIP	97.84	95.35	97.42	92.64	96.96	92.89
RTLNP	95.97	91.32	96.71	91.14	95.61	91.77
LTCP	90.21	86.30	88.25	81.28	87.64	89.10
FDLBP	97.91	96.60	**98.03**	93.91	96.63	94.17

**Table 4:** Accuracy (%) on TexLensDB and ColLensDB for N=1 and N=5 training images per class. The table includes the absolute gain in percentage points (pp), the Mean Standard Deviation (Mean Std), and the Global Average Performance (GAP).

Method	TexLensDB			ColLensDB			Mean Std	GAP
	1-Shot	5-Shot	Gain	1-Shot	5-Shot	Gain		
DNT-MQP	75.06	94.29	+19.23	66.66	89.23	+22.57	4.01	81.31
RALBGC	71.55	92.37	+20.82	68.85	87.92	+19.07	3.61	80.17
ARCSLBP	55.85	82.94	+27.09	62.99	82.33	+19.34	4.00	71.03
ARCSLBPrn	64.36	91.34	+26.98	63.04	85.53	+22.49	3.70	76.07
RGP	71.08	91.59	+20.51	78.73	93.97	+15.24	3.97	83.84
WMLBP	64.42	90.17	+25.75	52.94	76.79	+23.85	4.40	71.08
QS-LBP	75.54	94.63	+19.09	69.14	90.91	+21.77	3.44	82.56
MBM-LBP	58.13	80.20	+22.07	52.06	73.36	+21.30	3.97	65.94
TDLBP	75.48	94.69	+19.21	65.57	86.64	+21.07	3.49	80.60
SPALBP	58.28	82.52	+24.24	56.79	80.58	+23.79	4.20	69.54
MTSP	71.47	92.60	+21.13	65.50	87.24	+21.74	3.84	79.20
LNGP	52.40	78.42	+26.02	42.52	59.13	+16.61	3.77	58.12
CLTP	66.84	88.55	+21.71	51.09	74.77	+23.68	3.85	70.31
WDMBP	49.25	72.72	+23.47	40.79	58.51	+17.72	3.81	55.32
RCSP	78.43	95.31	+16.88	70.94	92.09	+21.15	3.40	84.19
ARLBP	80.30	96.11	+15.81	69.37	89.78	+20.41	3.34	83.89
RFD	74.07	94.91	+20.84	62.32	85.82	+23.50	3.71	79.28
DNTPDS	81.46	96.28	+14.82	72.10	90.64	+18.54	3.28	85.12
Hess-ACS-LBP	75.21	94.30	+19.09	66.02	87.20	+21.18	3.93	80.68
OD-LBP	77.85	95.65	+17.80	68.13	88.21	+20.08	3.47	82.46
MDGMMLTP	76.88	95.33	+18.45	69.49	91.52	+22.03	3.46	83.31
LGONBP	59.95	86.04	+26.09	63.46	89.00	+25.54	4.25	74.61
LCCMSP	71.85	92.45	+20.60	69.60	88.98	+19.38	3.54	80.72
LBP	70.76	91.16	+20.40	62.45	80.07	+17.62	3.46	76.11
PGMO-MSTP	72.82	93.67	+20.85	69.67	91.19	+21.52	3.78	81.84
SBP	59.89	86.98	+27.09	54.04	78.16	+24.12	4.06	69.77
LETRIST	71.04	93.25	+22.21	62.51	86.64	+24.13	4.42	78.36
PLGF	60.40	87.88	+27.48	50.36	75.30	+24.94	3.47	68.49
LDTP	68.94	93.13	+24.19	70.10	89.87	+19.77	3.81	80.51
nLBPd	64.86	90.87	+26.01	68.12	88.64	+20.52	3.70	78.12
LOOP	85.71	96.57	+10.86	73.81	84.07	+10.26	3.18	85.04
LQCH	53.90	85.09	+31.19	56.02	80.80	+24.78	4.49	68.95
PKLBP	75.92	94.31	+18.39	65.79	87.20	+21.41	3.54	80.81
CSQP	83.18	95.98	+12.80	82.37	91.76	+9.39	2.97	88.32
QxLBP	60.44	81.13	+20.69	53.82	76.53	+22.71	4.08	67.98
SWOBP	30.03	52.40	+22.37	70.88	90.98	+20.10	4.10	61.07
MCLBP	49.11	70.06	+20.95	62.70	87.20	+24.50	4.46	67.27
SSN	51.25	74.98	+23.73	56.41	80.62	+24.21	4.01	65.82
LQPAT	71.36	92.78	+21.42	68.61	88.09	+19.48	3.47	80.21
LDEBP	54.26	74.65	+20.39	40.87	56.22	+15.35	4.36	56.50
ILQP	71.06	92.56	+21.50	66.60	89.71	+23.11	3.92	79.98
DTLBP	71.76	92.87	+21.11	62.92	84.66	+21.74	3.60	78.05
MRELBP	61.59	87.82	+26.23	67.83	89.27	+21.44	3.62	76.63
dLBPa	70.26	92.18	+21.92	65.28	88.55	+23.27	3.47	79.07
LDZP	72.49	92.72	+20.23	67.66	85.22	+17.56	3.67	79.52
MCLPB	73.09	94.50	+21.41	59.11	83.19	+24.08	3.61	77.47
LNIP	64.13	89.80	+25.67	52.73	74.41	+21.68	3.63	70.27
FLNIP	76.23	94.16	+17.93	70.80	89.98	+19.18	3.36	82.79
RTLNP	64.54	90.83	+26.29	57.23	82.96	+25.73	4.12	73.89
LTCP	63.94	84.60	+20.66	63.65	81.71	+18.06	3.58	73.48
FDLBP	77.47	95.53	+18.06	69.97	91.50	+21.53	3.35	83.62

**Table 5:** Comparison of the recognition rates obtained with the reference 1-NN classifier (L1 distance) and the evaluated SVM classifiers for the ten highest-ranked texture descriptors.

Texture Descriptor	1-NN (L1)		Linear SVM		Polynomial SVM		RBF SVM	
	TexLensDB	ColLensDB	TexLensDB	ColLensDB	TexLensDB	ColLensDB	TexLensDB	ColLensDB
RCSP	98.30	96.59	97.25	96.27	97.31	95.80	97.21	96.34
MDGMMLTP	98.20	96.76	97.60	95.61	97.55	95.44	97.60	95.62
QS-LBP	98.24	96.07	97.11	95.85	97.11	95.74	97.10	95.86
DNTPDS	98.42	95.66	97.98	95.93	97.99	96.02	97.98	95.92
FDLBP	97.91	96.60	97.26	96.48	97.26	96.07	97.26	96.47
ARLBP	98.33	95.16	97.63	94.75	97.80	94.26	97.64	94.80
FLNIP	97.84	95.35	96.54	94.73	96.49	94.21	96.53	94.71
PGMO-MSTP	97.08	96.22	97.10	94.95	97.23	94.63	97.12	94.98
RGP	96.45	97.29	97.09	96.26	97.16	95.97	97.14	96.27
OD-LBP	97.86	94.61	97.33	95.39	97.36	95.15	97.33	95.38

**Table 6:** Ranking results obtained using the Wilcoxon-based ranking test according to the normalized number of victories reached by each evaluated texture operator on the two used datasets.

Ranking 1-NN	Texture Descriptor	Victories/Comparisons	Dimension
1	RCSP	0.93000	768
2	MDGMMLTP	0.92000	1024
3	QS-LBP	0.89000	512
4	DNTPDS	0.88000	2048
5	FDLBP	0.85000	4096
6	ARLBP	0.83000	1792
7	FLNIP	0.77000	1024
8	PGMO-MSTP	0.75000	4096
9	RGP	0.71000	1728
10	OD-LBP	0.70000	768
11	DNT-MQP	0.68000	384
12	CSQP	0.68000	256
13	RFD	0.65000	4608
14	TDLBP	0.62000	512
15	LCCMSP	0.60000	2046
16	LDTP	0.60000	1022
17	PKLBP	0.57000	1280
18	ILQP	0.57000	1024
19	LETRIST	0.56000	413
20	Hess-ACS-LBP	0.55000	400
21	LGONBP	0.55000	1404
22	LQPAT	0.55000	512
23	MCLPB	0.51000	6962
24	MRELBP	0.50000	800
25	dLBPa	0.50000	256
26	RALBGC	0.46000	1022
27	LOOP	0.45000	256
28	MTSP	0.44000	256
29	nLBPd	0.44000	256
30	ARCSLBPn	0.42000	4096
31	SWOBP	0.39000	3500
32	LDZP	0.39000	354
33	DTLBP	0.37000	256
34	RTLNP	0.35000	1024
35	MCLBP	0.33000	531
36	LQCH	0.27000	6001
37	LBP	0.26000	256
38	SBP	0.23000	400
39	WMLBP	0.22000	2200
40	SPALBP	0.21000	200
41	LNIP	0.21000	512
42	ARCSLBP	0.20000	256
43	PLGF	0.20000	512
44	CLTP	0.17000	512
45	LTCP	0.17000	256
46	SSN	0.16000	1134
47	QxLBP	0.12000	312
48	MBM-LBP	0.09000	512
49	LNGP	0.05000	256
50	WDMBP	0.05000	256
51	LDEBP	0.02000	64

**Table 7:** Comparison between the best handcrafted texture descriptors and pretrained deep feature extractors. Bold values indicate the best recognition result within the corresponding method category/dataset.

Method	Category	TexLensDB (%)	ColLensDB (%)
**Handcrafted texture descriptors**			
RGP	Handcrafted	**98.57**	98.71
DNTPDS	Handcrafted	98.45	96.21
RCSP	Handcrafted	98.52	96.52
QS-LBP	Handcrafted	**98.66**	96.07
MDGMMLTP	Handcrafted	98.20	96.76
**Pretrained deep feature extractors**			
ResNet18	CNN	98.14	99.60
MobileNetV3-Large	CNN	**98.56**	**99.63**
EfficientNet-B0	CNN	99.38	99.56
ViT-B16	Transformer	98.43	99.11
ViT-B32	Transformer	98.61	99.31
Swin-T	Transformer	95.73	99.51
Swin-B	Transformer	98.14	99.15
MaxViT-T	Transformer	97.17	96.66

## Data Availability

The datasets and resources supporting the findings of this study are available from the corresponding author upon reasonable request.
